# Telomere to telomere flax (*Linum usitatissimum* L.) genome assembly unlocks insights beyond fatty acid metabolism pathways

**DOI:** 10.1093/hr/uhaf127

**Published:** 2025-05-07

**Authors:** Jianyu Lu, Hanlu Wu, Fu Wang, Jinxi Li, Yifei Wang, Qian Zhao, Yingping Wang, Xiaonan Wang, Xiujuan Lei, Ruidong Sun, Jun Zhang, Aisheng Xiong, Michael K Deyholos, Jian Zhang

**Affiliations:** Faculty of Agronomy, Jilin Agricultural University, Changchun 130118, China; International Biotechnology Laboratory for Fiber Plants, Changchun 130118, China; Faculty of Agronomy, Jilin Agricultural University, Changchun 130118, China; International Biotechnology Laboratory for Fiber Plants, Changchun 130118, China; Faculty of Agronomy, Jilin Agricultural University, Changchun 130118, China; International Biotechnology Laboratory for Fiber Plants, Changchun 130118, China; Faculty of Agronomy, Jilin Agricultural University, Changchun 130118, China; International Biotechnology Laboratory for Fiber Plants, Changchun 130118, China; Faculty of Agronomy, Jilin Agricultural University, Changchun 130118, China; International Biotechnology Laboratory for Fiber Plants, Changchun 130118, China; Faculty of Agronomy, Jilin Agricultural University, Changchun 130118, China; International Biotechnology Laboratory for Fiber Plants, Changchun 130118, China; College of Chinese Medicinal Materials, Jilin Agricultural University, Changchun 130118, China; Institute of Fiber Plants, Heilongjiang Academy of Science, Daqing, China; College of Chinese Medicinal Materials, Jilin Agricultural University, Changchun 130118, China; Faculty of Agronomy, Jilin Agricultural University, Changchun 130118, China; International Biotechnology Laboratory for Fiber Plants, Changchun 130118, China; Faculty of Agronomy, Jilin Agricultural University, Changchun 130118, China; International Biotechnology Laboratory for Fiber Plants, Changchun 130118, China; College of Horticulture, Nanjing Agricultural University, Nanjing, Jiangsu 210095, China; International Biotechnology Laboratory for Fiber Plants, Changchun 130118, China; Department of Biology, University of British Columbia, Okanagan, Kelowna, BC V5K1K5, Canada; Faculty of Agronomy, Jilin Agricultural University, Changchun 130118, China; International Biotechnology Laboratory for Fiber Plants, Changchun 130118, China; Department of Biology, University of British Columbia, Okanagan, Kelowna, BC V5K1K5, Canada

## Abstract

One of China’s most important resources is flax (*Linum usitatissimum* L.), an ancient crop with significant nutritional and therapeutic benefits. Despite its importance, existing flax reference genomes remain incomplete, with many unassembled sequences. Here, we report a gapless 482.51 Mb telomere-to-telomere (T2T) flax genome assembly, predicting 46 634 genes, of which 42 805 were functionally annotated. Repetitive sequences constitute 60.05% of the genome, and we identified 30 telomeres and 15 centromeres across the chromosomes. Whole-genome duplication (WGD) events were detected at approximately 11.5, 53.5, and 114 million years ago (MYA) based on synonymous substitution rates (Ks). The T2T assembly enabled the reconstruction of the fatty acid metabolic pathway, identifying 49 related genes, including six newly annotated ones. Furthermore, genomic colocalization was observed between fatty acid metabolism pathway-related genes and transposable elements, suggesting that functional differentiation of these genes in flax evolution may have occurred through transposon-mediated duplication events. Phylogenetic analysis of *SAD* and *FAD* gene families revealed that *FAD* genes segregate into *FAD2* and *FAD3/7/8* subfamilies. Gene structure and motif analyses demonstrated conserved exon–intron architectures and motif organization within each phylogenetic clade of *SAD* and *FAD* genes. Promoter region characterization identified numerous *cis*-acting elements responsive to phytohormones (MeJA and abscisic acid) and abiotic stresses (low temperature and anaerobic induction) in both *SAD* and *FAD* genes. Our knowledge of the evolution of the flax genome is improved by this excellent genome assembly, which also offers a strong basis for enhancing agricultural attributes and speeding up molecular breeding.

## Introduction

A member of the Linaceae family, flax (*Linum usitatissimum* L.) is an annual herbaceous plant that has long been grown all over the world, particularly in temperate regions. It is also one of China’s major economic crops [1, 2]. Flax is classified into three types based on its use: oilseed, fiber, and dual-purpose for both oil and fiber [3]. Flaxseed has long been regarded as a valuable source of nutrients for both humans and animals [4]. Flaxseed is also an important industrial crop, with its oil being used in painting, chemicals, and dyes [5]. Nutrients including lignans and dietary fiber are abundant in flaxseeds, containing 40%–50% oil, ~73% polyunsaturated fatty acids, and ~50% α-linolenic acid (C18:3), Omega-3 fatty acids, which are essential for human health, are abundant in it [6]. The bioactivity of ω-3 polyunsaturated fatty acids (PUFAs) has been shown to help prevent inflammation, diabetes, cancer, and cardiovascular diseases [7, 8]. Therefore, flax is a highly promising economic crop with significant industrial value.

Understanding molecular regulation is essential for revealing the mechanisms behind fatty acid production [9]. Previous studies have identified key enzymes in the route leading to the synthesis of fatty acids, including fatty acid desaturases and elongases [10]. A crucial phase in the production of plant oils is the progressive desaturation of fatty acids, which establishes the proportion of unsaturated to saturated fatty acids [11]. Enzymes called fatty acid desaturases are in charge of adding double bonds to fatty acid hydrocarbon chains [12]. The membrane-bound proteins known as FADs are made up of three highly conserved histidine-box motifs that are necessary for enzymatic activity [13]. In contrast, SADs represent the only known family of soluble desaturases, with the ability to convert 18:0-ACP to 18:1-ACP [14]. In flax, two homologous genes, *sad1* and *sad2*, exhibit distinct expression patterns across different tissues [15]. Although the functions of genes encoded by some restriction enzymes have been preliminarily confirmed [16–18], there are still many gaps that need to be filled. The incomplete genetic information of flax has hindered the study of its molecular mechanisms and led to the incomplete characterization of other important genes related to restriction enzymes.

As high-throughput sequencing technology has advanced, genome assembly has experienced a radical change, enabling the construction of complete, gapless telomere-to-telomere (T2T) genomes [19, 20]. T2T genomes encompass the entire genomic information of a species, representing the ultimate goal of genome assembly. This approach effectively eliminates mapping errors, enhances the accuracy of variant detection, facilitates the identification of missing genes and genetic information, and enables the study of centromere and telomere evolutionary histories [19, 21]. So far, T2T genomes of multiple species have been published, such as *Arabidopsis* [22], *Glycine max* [23], *Zea mays* [24], *Fragaria vesca* [25], *Daucus carota* [26], *Vitis vinifera* [27], *Vaccinium duclouxii* [28], and *Dianthus caryophyllus* [29]. However, there have been no reports of T2T assembly of flax. Currently, the most commonly used genome assemblies of flax are the CDC Bethune genome and Longya10, which were obtained through Illumina sequencing from the Canadian variety CDC and the Chinese Xinjiang variety Longya10 [30–32]. Although high-throughput second-generation sequencing platforms have significantly improved genome quality, assembled genomes still contain numerous unresolved regions and lack completeness. Telomeres are largely unknown, as highly conserved microsatellite sequences positioned at eukaryotic chromosome ends [33] and are crucial for maintaining cell mitosis [34]. As a result, a gapless, high-quality flax genome would theoretically support breeding efforts, provide insight into the evolutionary history of centromeres and telomeres, and greatly enhance studies on species evolution.

In this study, the oil-fiber dual-purpose flax variety Gaosi from the Heilongjiang Academy of Agricultural Sciences was selected as plant material. By combining Hi-C technology, PacBio HiFi data, and ONT ultralong (UL) reads, the first fully gapless T2T genome of flax Gaosi was assembled. All telomere and centromere regions of the flax genome were identified, and comparative genomic analyses were conducted. Additionally, homologous alignment was used to reannotate fatty acid metabolic pathway genes in the flax T2T genome, with a further focus on analyzing key fatty acid desaturase (*FAD*) and stearoyl-ACP desaturase (*SAD*) gene family members. The findings of this study address gaps in flax genome research and provide a theoretical foundation for cultivar breeding, genetic improvement, and investigations into molecular mechanisms.

## Results

### Assembly and annotation of Gaosi flax T2T genome

**Figure 1:**
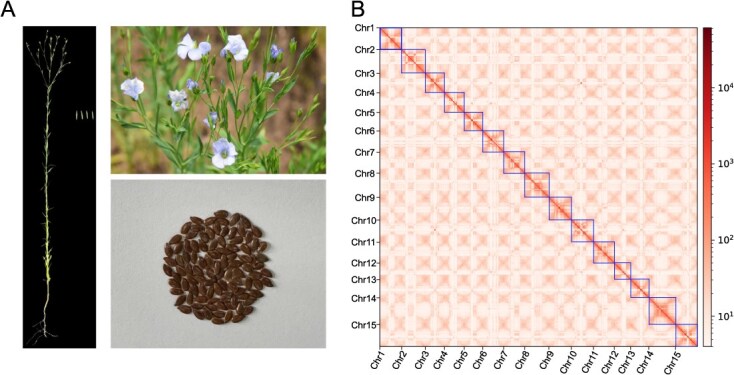
Phenotypic characteristics and gapless genomic features of flax. (A) Phenotypes of different organs in flax ‘Gaosi’. (B) Hi-C interaction heatmap of flax chromosomes.

The flax genome’s size and heterozygosity were assessed using K-mer analysis and second-generation sequencing. Analysis of the sample’s depth–frequency distribution at K = 19 showed that the genome of Gaosi flax is roughly 460.13 Mb in size, with a heterozygosity rate of 0.34% (Table S1, Fig. S1). Genome assembly was performed using 15.73G UL ONT sequencing data, 21.95G HiFi sequencing data, 20.86G second-generation sequencing data, and 55.91G Hi-C sequencing data (Tables S1–S5), and three assembly strategies were employed (HiFi+UL ONT, HiFi and UL ONT) (Table S6). Twelve gaps were found when the contigs were anchored to 15 pseudomolecules using Hi-C sequencing data (Table S7). These gaps were filled using UL-ONT data, resulting in the finalized T2T-level Gaosi flax genome. The genome size increased from 479.046 Mb to 482.51 Mb, covering all 15 chromosomes. The read mapping locations are presented in Fig. S2. Compared to the previously reported Longya10 reference genome, the Gaosi T2T genome assembly showed significant improvements, with an increase of ~176.1 Mb in size and a 32.9 Mb enhancement in Contig N50. Additionally, 15 centromeres and 30 telomeres were identified. In comparison to the previous version, the Gaosi T2T genome’s guanine and cytosine (GC) content was somewhat lower (Table 1). As shown in Fig. 1B, the Hi-C heatmap of the assembled T2T chromosomes demonstrates strong interaction intensity across all chromosomes, with no abnormal interaction signals observed. This validates the great precision of the Hi-C anchoring results and the flax T2T genome assembly. The assembled genome was aligned with reads from second-generation sequencing, HiFi, and UL ONT to evaluate genome consistency (Table S7). The second-generation sequencing readings have a coverage rate of 99.81% and a mapping rate of 99.71%. The HiFi readings showed a 99.86% coverage percentage and a 99.98% mapping rate (Table S8). The mapping rate of the UL ONT reads was 100%, having a 99.96% coverage percentage. According to the results of the BUSCO analysis, which assessed the genome integrity of Gaosi T2T, the assembly of the genome was complete since 97.3% of conserved genes had complete matches, comprised of 1107 multiple-copy homologous genes and 463 single-copy homologous genes (Table S9). According to the K-mer data, the Gaosi T2T genome’s QV value was 47.38, and the QV values of the 15 chromosomes ranged from 45.11 to 49.01 (Table S10), proving that the assembled Gaosi T2T genome had high accuracy.

**Table 1:** Assembly statistics of flax genome.

**Parameter**	**Longya10**	**CDC Bethune genome**	**Gaosi T2T**
Genome size (Mb)	306.4	316.2	482.51
Contig N50 (Mb)	0.21	0.023	33.03
Number of contigs	6319	26 086	15
Number of telomeres			30
Number of centromeres			15
Length of gaps (Mb)	5.8	46.58	0
GC content (%)	39	39.5	37.6
BUSCOs of genome (%)	91.53	93.5	97.3

**Figure 2:**
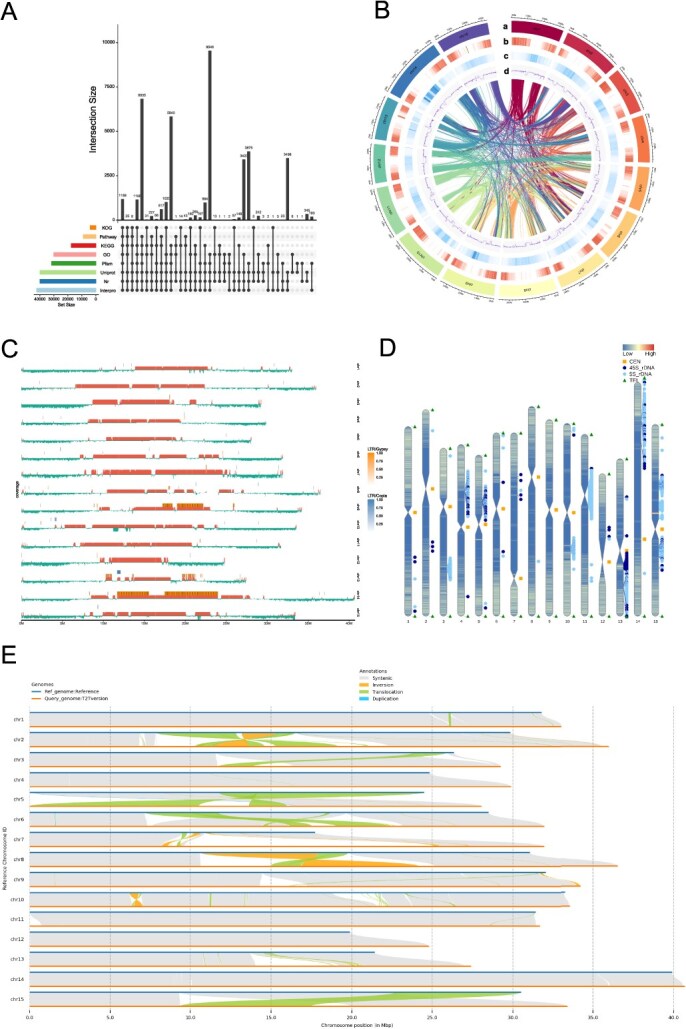
High-quality T2T genome assembly of flax. (A) Flax gene functional annotation. (B) Features of the flax T2T genome: (a) assembly of chromosomes, (b) gene density, (c) density of gene duplication, (d) GC content. The lines inside the circle represent collinear gene pairs. (C) Distribution of tandem repeat sequences and LTR elements in the flax genome. (D) Predicted centromeric and telomeric regions of the T2T genome. (E) Structural variations between the Longya10 genome and the T2T genome.

The protein structure of the flax genome was determined through *de novo* prediction, homology comparison (comparing with the genomes of *Arabidopsis thaliana*, *Linum tenue*, and *L. usitatissimum*), and transcriptome prediction, and the final data were obtained after redundancy removal (Table S11). The Gaosi genome was expected to include 192 875 introns, 239 509 exons, and 46 634 genes in total (Table S12). Exons, introns, mRNA, and coding sequences (CDS) had average lengths of 280.53, 263.78, 2531.75, and 1151.92 bp, respectively. On average, there were 5.14 exons per gene. A comparative study of gene length between flax and related species found that the gene frequency and intron length distribution of the flax genome are similar to those of other plants, but there are some differences in gene length and CDS length compared to the *A. thaliana* genome (Fig. S3). The protein sequences that the predicted genes encoded were aligned using a number of protein databases (Table S12), of which 42 805 (91.79%) were matched with the database and obtained at least one annotation, including 42 153 (90.39%) in the Interpro database, 39 864 (85.48%) in the nr database, 39 824 (85.40%) in the Uniprot database, 31 531 (67.61%) in the Pfam database, 29 975 (64.28%) in the Gene Ontology (GO) database, 17 460 (37.44%) in the Kyoto Encyclopedia of Genes and Genomes (KEGG) database, and 3890 (8.34%) in the Eukaryotic Clusters of Orthologous Groups (KOG) database (Fig. 2A). A total of 289.72 Mb of repeated sequences, or 60.05% of the entire flax genome, were obtained by annotating the repetitive sequences in the T2T genome of flax using a variety of prediction techniques. The predominant type in the flax genome is long terminal repeats (LTR), accounting for 20.3%, including LTR-Gypsy (6.75%) and LTR-Copia (4.21%) (Fig. 2C) (Table S13). The repetitive sequences also include six other types (CACTA, PIF-Harbinger, Tc1, hAT, LINE, and Helitron), which are unevenly distributed on chromosomes (Fig. S4).

Based on structural characteristics, the flax genome contained 170 miRNAs, which made up 0.0043% of the genome and had an average length of 122 bp. Furthermore, genomic annotation identified three classes of non-coding RNAs as documented in Table S14: 1,255 tRNAs averaging 75 bp in length (0.0195% of the genome), 759 snRNAs with a mean length of 121 bp (0.0191% genomic proportion), and 23,130 rRNAs averaging 141 bp (0.6741% of the genome).

### Telomere and centromere identification

Based on the gene density, repetitive sequence density, and GC content of the flax T2T genome, a Circos diagram of the genome was drawn (Fig. 2B), and it was found that most of the coding genes were mainly distributed at the ends of the chromosome (Fig. 2C and D). A comparison with the genome of flax Longya10 revealed that two different types of flax genomes underwent structural rearrangements including 50 translocations, 15 inversions, and 3 duplications (Fig. 2E). The T2T genome of flax fills many gaps in previous versions of the genome. All 30 telomeres on 15 chromosomes were identified based on the telomeric repeat sequences (CCCTAAA and TTTAGGG) (Table S15). With an average of 1863 and 2188, respectively, the number of downstream telomeric repeats in the flax T2T genome spans from 1159 to 2785, whereas the number of upstream telomeric repeats varies from 1045 to 2489. Low gene density sequences and continuous high- and short-tandem repeats were used to determine the centromeric regions of flax, and all 15 centromeres were identified on 15 chromosomes (Table S16). The centromere boundaries of its flax genome are between 136 477 and 8 416 650 bp, with an average length of 3567468.93 bp. These regions were excluded from earlier versions of the flax genome.

### Comparative genomics and evolutionary analysis

**Figure 3:**
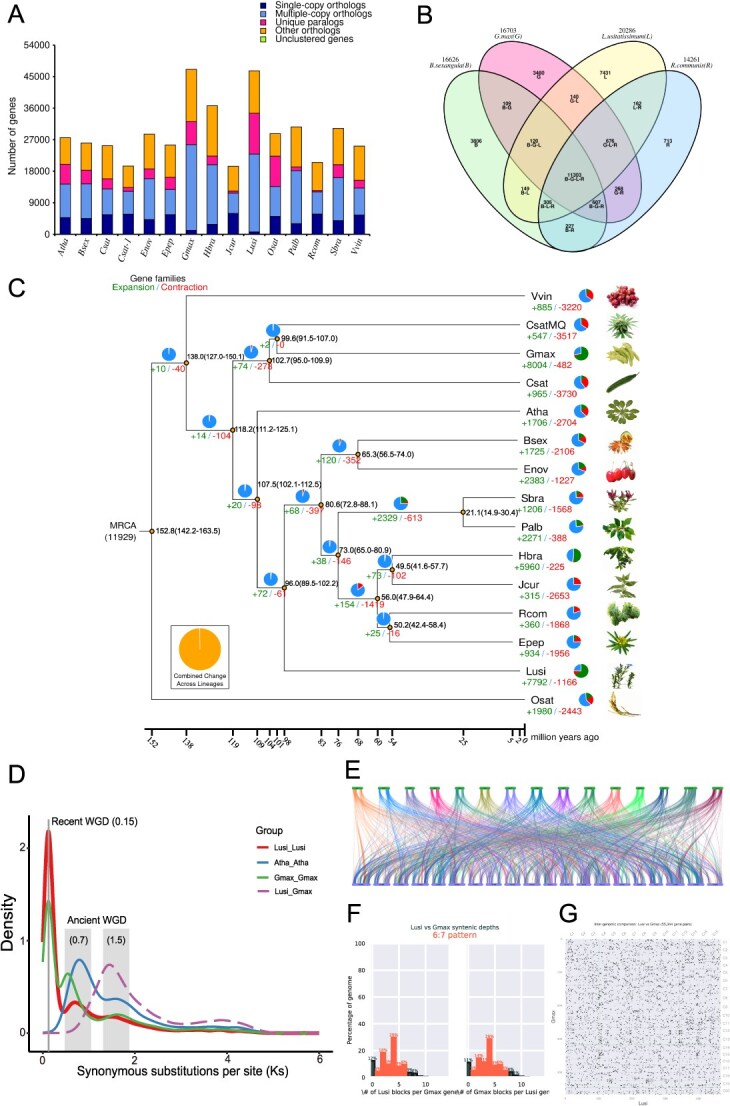
Examination of the flax genome’s evolution. (A) The number of genes that are homologous between 14 species and flax. (B) Four species’ gene families were analyzed using clustering. The number indicates how many gene families there are, while the letters in parenthesis stand for the species’ acronyms, the letter string represents the common gene families between species, and a single letter represents the unique gene families within a species. (C) Evolutionary tree showing the divergence periods and phylogenetic relationships of 15 species. During the evolutionary process, ‘+’ and ‘−’ indicate the expansion and contraction of gene family numbers, respectively. (D) Display of synonymous substitution numbers (Ks) for each synonymous locus. (E) Collinearity analysis of flax and soybean genomes. Different colors represent collinearity between different chromosomes. (F) Syntenic depth ratio analysis of flax and soybean. (G) Scatter plots comparing the genomes of flax and soybean.

The genomes of 15 species (*Vvin*, *V. vinifera*; *CsatMQ*, *Cannabis sativa*; *Gmax*, *G. max*; *Csat*, *Cucumis sativus*; *Atha*, *A. thaliana*; *Bsex*, *Bruguiera sexangula*; *Enov*, *E. novogranatense*; *Sbra*, *Salix brachista*; *Palb*, *Populus alba*; *Hbra*, *Hevea brasiliensis*; *Jcur*, *Jatropha curcas*; *Rcom*, *Ricinus communis*; *Epep*, *Euphorbia peplus*; *Osat*, *Oryza sativa*) were employed for single-copy and multicopy gene enrichment, homologous gene identification, and gene family clustering analysis (Fig. 3A). Across the 15 species, 48 883 homologous gene families containing 437 543 genes were identified. Among these, 7883 gene families and 227 340 genes were shared across all species. 11 633 genes from 7103 distinct gene families were found in flax (Table S17). The GO and KEGG pathway enrichment was used to perform functional assessments of genes specific to flax. Flax-specific genes were linked to defense response, signal transduction, iron ion binding, and oxidoreductase activity, which involves the incorporation or reduction of molecular oxygen, according to the GO enrichment study. Genes unique to flax were mostly associated with pathways such as the metabolism of starch and sucrose and plant–pathogen interaction, according to KEGG pathway enrichment study, phenylpropanoid biosynthesis, and the biosynthesis of various secondary metabolites (Fig. S5). Comparative clustering of gene families was performed using closely related species *B. sexangula*, *G. max*, and *R. communis* (Fig. 3B). The results showed that these four species had 11 303 gene families in common. Flax shared 11 877, 12 239, and 12 446 gene families with *B. sexangula*, *G. max, and R. communis*, respectively. Notably, flax contained 7431 unique gene families.

Six hundred eighty single-copy genes were used to create a phylogenetic tree, with *V. vinifera* and *O. sativa* acting as outgroups (Fig. 3C). The results indicated that the Brassicaceae and Linaceae families shared a common ancestor and diverged between 102.1 and 112.5 million years ago (MYA). Subsequently, flax diverged from the Brassicaceae lineage ~89.5–102.2 MYA. Its earlier divergence from other species within the Malpighiales order (*B. sexangula*, *E. novogranatense*, *S. brachista*, and *P. alba*) further supports the notion that flax is an ancient crop. Using CAFE4.2 software, the growth and shrinkage of flax gene families were forecasted. The results revealed that flax exhibited 7792 expanded gene families, encompassing 11 619 genes, and 1166 contracted gene families, comprising 1454 genes. The increased gene families were mainly linked to auxin response, according to GO and KEGG pathway enrichment analyses, lignin catabolic process, mRNA surveillance pathway, and oxidative phosphorylation. On the other hand, the contracted gene families were primarily associated with the endoplasmic reticulum’s protein processing, DNA integration, nucleic acid binding, and polysaccharide binding (Figs S6 and S7).

The number of synonymous substitutions per synonymous site (Ks) was used to estimate the date of large-scale duplication events, either between species or within the same species (Fig. 3D). A shared whole-genome duplication (WGD) event among dicotyledonous plants was observed in both *L. usitatissimum* and *legumes* (*G. max* and *Arachis hypogaea*), with a Ks value of ~1.5. When the Ks values were ~0.15, 0.7, and 1.5, *L. usitatissimum* underwent three independent WGD events. Flax undergoes whole-genome replication events at ~11.5, 53.5, and 114 MYA, respectively. The fact that the entire genome replication event that took place at 114 MYA was so close to the point at which the *Brassicaceae* and *Linaceae* began to differentiate suggests that it played a significant role in the two species’ separation. A comparison was made between the chromosome evolution of flax and its closely related species (*G. max*), and large-scale collinear gene pairs were detected between them (Fig. 3E and G). In addition, the overall synthesis depth ratio of collinearity analysis is 6:7, indicating that both plants have experienced the same WGD event. These research findings provide new clues for understanding the evolution of flax chromosomes.

### Analysis of fatty acid metabolism pathways in flax

**Figure 4:**
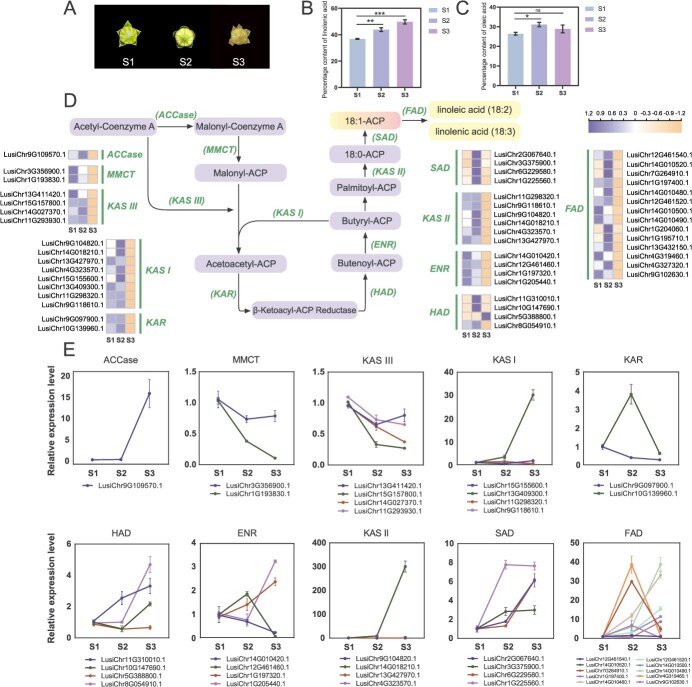
Metabolic pathways of flax fatty acids. (A) Flaxseed during various stages of development: Fruit tissues at 10, 20, and 30 days following flowering correspond to S1, S2, and S3 stages, respectively. (B) The proportion of total fatty acids to linolenic acid. (C) The proportion of total fatty acids that are oleic acid. Asterisks indicate statistically significant differences between treatment and control groups determined by Student’s *t*-test (*P < 0.05; **P < 0.01; ***P < 0.001). (D) Flax fatty acid metabolic pathways and related gene expression levels. The heatmap illustrates gene expression across S1–S3 developmental stages. (E) Relative gene expression levels associated with flax fatty acids. Data are shown as mean ± SE (*n* = 3).

Following blooming, the levels of oleic and linolenic acids in flax fruits were assessed at various developmental phases (Fig. 4A–C). The results showed that the linolenic acid content exhibited an increasing trend during fruit development, reaching its peak at the S3 stage (30 days after flowering), where stearic acid, palmitic acid, oleic acid, linolenic acid, and linoleic acid were among the fatty acids that comprised 49.72% of the total amount. During fruit development following flowering, the oleic acid concentration first increased and subsequently decreased, reaching its peak at the S2 stage (20 days after flowering), where it accounted for 31.08% of the total fatty acids (Table S18).

Using the flax T2T genome, we mapped the fatty acid metabolic pathway and investigated its regulatory mechanisms (Fig. 4D). Through rigorous screening and homology alignment, we were able to identify 49 structural genes in the fatty acid metabolism pathway (Table S19). Compared with the Longya10 genome, 43 genes can be mapped to it, and the flax T2T genome has six newly identified genes. The majority of the structural genes found were found on chromosomes 1, 9, and 14, which contained 7, 8, and 7 genes, respectively, with gene lengths ranging from 483 to 1692 bp. We also identified that the *acetyl-CoA carboxylase* (*ACCase*) gene is a single-copy gene, whereas other genes involved in fatty acid biosynthesis pathways have multiple copies located on the same or different chromosomes. In flax, four genes were found to belong to the *SAD* family, and 14 genes were classified under the *FAD* family, both of which encode key fatty acid desaturation and elongation enzymes. The genes for β-ketoacyl-ACP synthase (KAS), β-Hydroxyacyl-ACP Dehydratase (HAD), and acyl-ACP reductase (ENR) were also discovered to have substantially high copy counts. We discovered transposons that overlap with genes involved in fatty acid production based on the annotated repetitive sequences in the flax T2T genome. The results revealed various types of transposons located near *MCAT*, *KAS III,* five *KAS I/II*, two *ENR*, three *SAD*, and seven *FAD* genes. Among these, overlapping transposons included DNA/MULE-MuDR, DNA/PIF-Harbinger, LINE/L1, LTR/Copia, DNA/hAT-Ac, LTR/ERV1, and several unknown categories (Table S20).

To explore the potential functions of genes implicated in the fatty acid metabolic pathway during flax fruit development, we employed RNA-seq data to analyze the expression patterns of all identified genes (Fig. 4D). The majority of genes were found to be highly expressed in flowers 10 days (51.02%) and 20 days (77.55%) following blossoming, according to the data. Thirty days after flowering, the fruit showed high expression of only one gene (*LusiChr5G388800.1*). In order to acquire precise expression trends, we then used quantitative real-time polymerase chain reaction (qRT-PCR) to validate the relative expression levels of these genes (Fig. 4E). According to the findings, the S3 stage was when the genes for acetyl-CoA carboxylase (ACCase), β-ketoacyl-ACP synthase I (KAS I), and β-ketoacyl-ACP synthase II (KAS II) were most highly expressed. Among them, *LusiChr13G409300.1* and *LusiChr14G018210.1* were the predominant expressed genes for *KAS I* and *KAS II*, respectively. The genes encoding malonyl-CoA-ACP transferase (MMCT) and β-ketoacyl-ACP synthase III (KAS III) exhibited a gradual decline in expression as seed development progressed. We inferred that *MMCT* and *KAS III* genes had their highest expression during the S1 stage. The transcript level of the *β-ketoacyl-ACP reductase* (*KAR*) gene increased initially and then decreased during development, with *LusiChr10G139960.1* identified as the predominantly expressed gene. Among the four *HAD* genes, the expression levels of three genes surged from the S2 to S3 stages, exhibiting similar expression patterns. In contrast, the four *ENR* genes displayed an opposite expression trend. For the *SAD* genes, two genes (*LusiChr1G225560.1* and *LusiChr3G375900.1*) were actively expressed during the S1 to S2 stages, while *LusiChr2G067640.1* and *LusiChr6G229580.1* were mostly manifested between the S2 and S3 phases. Among the genes for *FAD*, three genes (*LusiChr4G319460.1*, *LusiChr7G264910.1*, and *LusiChr1G197400.1*) showed a similar pattern of increasing expression followed by a decline. Meanwhile, the transcription levels of the other five genes gradually increased throughout development, reaching their highest levels at the S3 stage.

### Analysis of the SAD and FAD gene families associated with desaturase and fatty acid synthesis

**Figure 5:**
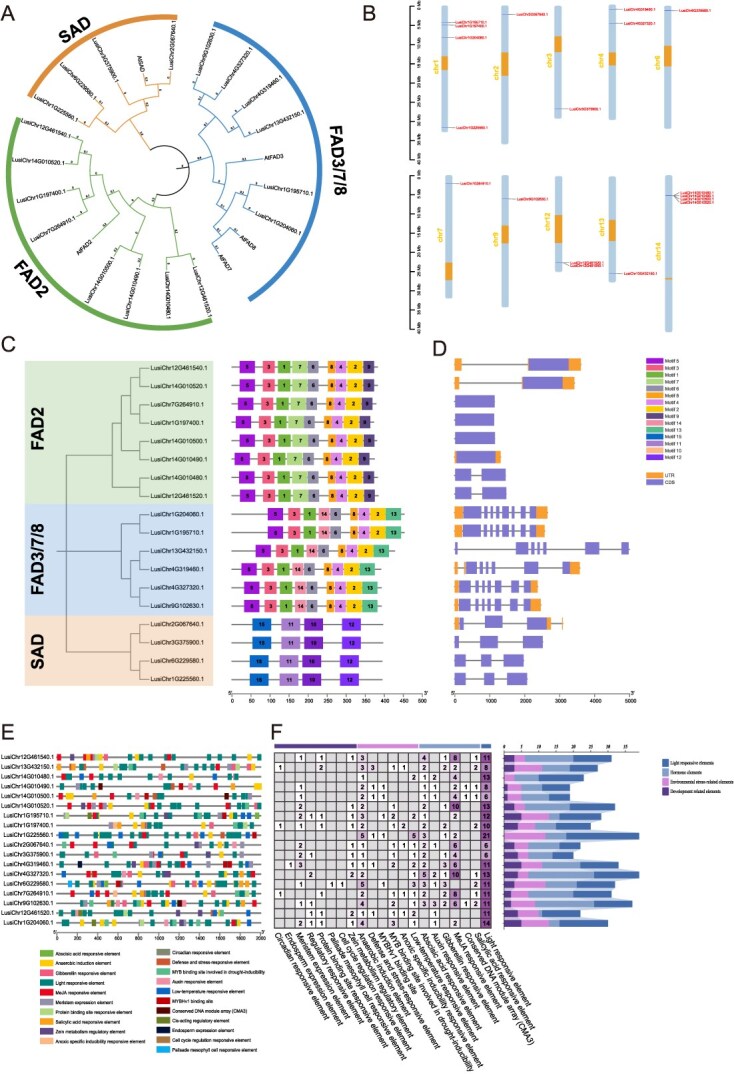
Identification of the gene families *SAD* and *FAD* associated with flax’s fatty acid metabolism. (A) Phylogenetic tree of SAD and FAD proteins in flax and *Arabidopsis*. (B) Examination of the chromosomes’ *SAD* and *FAD* genes. The orange square represents the centromere region. (C) *SAD* and *FAD* genes’ motif analysis. (D) *SAD* and *FAD* genes’ structural characterization. (E) Examination of the *SAD* and *FAD* genes’ *cis*-acting components. (F) A statistical examination of the quantity of *FAD* and *SAD* genes that are *cis*-acting.

Through homology alignment with the *Arabidopsis SAD* gene (*AT2G43710*) and *FAD* genes (*AT3G12120*, *AT3G11170*, *AT2G29980*, and *AT5G05580*), a total of four *SAD* genes and 14 *FAD* genes were identified in flax. *Arabidopsis* amino acid sequences (5 genes) and flax (18 genes) were used to construct a phylogenetic tree in order to better understand the phylogenetic links of the flax *SAD* and *FAD* gene families, resulting in a total of 23 genes. These genes were grouped into three subfamilies, designated as *SAD*, *FAD3/7/8*, and *FAD2* (Fig. 5A). The chromosomal localization of *SAD* and *FAD* genes in flax was determined based on the T2T genome. It was found that the 18 *SAD* and *FAD* genes were unevenly distributed across 10 chromosomes (Fig. 5B). Among them, chromosomes 1 and 14 harbored the highest number of *SAD* and *FAD* genes, with four genes each (22.22% of the total). This was followed by chromosomes 4 and 12, which each contained two *SAD* and *FAD* genes (11.11% of the total).

The MEME suite was used to analyze the amino acid sequences of 18 SAD and FAD proteins in order to visualize the protein structures of the flax *SAD* and *FAD* gene families. This analysis identified 15 conserved motifs, designated as motif1 to motif15 (Fig. 5C). Pfam analysis of motifs 1–15 identified motifs 1–5, 10–12, and 15 as linked to fatty acid desaturase activity (Table S21). In the *FAD2* and *FAD3/7/8* subfamilies, all genes contained motif1-motif6 and motif8. Additionally, four motifs (motif10–12 and motif15) were identified as unique to the *SAD* subfamily, while two motifs (motif7 and motif9) were specific to the *FAD2* subfamily. Similarly, two motifs (motif13 and motif14) were found to be exclusive to the *FAD3/7/8* subfamily. In conclusion, SAD and FAD proteins belonged to the same subfamily and had comparatively comparable motif kinds, quantities, and distributions. Exon–intron structure analysis of *SAD* and *FAD* genes showed exon counts ranging from 1 to 8 and intron counts from 0 to 7 (Fig. 5D). Notably, the genes *LusiChr1G204060.1* and *LusiChr1G195710.1* had the greatest amount of introns and exons, with nine and eight exons, respectively. To further understand the regulatory mechanisms of the *SAD* and *FAD* gene families in response to abiotic stress, promoters were predicted within the 2000-bp upstream regions of the *SAD* and *FAD* genes. After excluding common elements like the CAAT-box and TATA-box, 509 *cis*-acting elements were identified. Development-related, stress-responsive, hormone-related, and light-responsive elements were the four categories into which these components were divided (Fig. 5E and F). With 196 elements (38.5%), light-responsive elements were the most prevalent *cis*-acting elements in the *SAD* and *FAD* gene families, including GT1-motif and G-box. The second most abundant category was hormone-responsive elements, comprising 160 *cis*-acting elements (31.43%), which included MeJA-responsive elements (TGACG-motif and CGTCA-motif) and abscisic acid-responsive elements (ABRE). The third category included 101 components (19.84%) associated to environmental stress, including low-temperature responsive elements (LTR) and anaerobic induction elements (ARE). Fifty-two elements (10.22%) in the fourth group were associated to development and included zein metabolism control elements (O2-site) and meristem expression elements (CAT-box).

## Discussion

Flax is an important economic crop, rich in dietary fiber and lignans, among other nutrients. Among its many benefits, one of the important omega-3 fatty acids is alpha-linolenic acid (ALA), making it a crucial source for human health [6]. The flax genome has been put together at the chromosomal level in earlier research, although the materials used were from the oilseed variety Longya10 [31]. Given the distinct evolutionary and domestication histories between oilseed and fiber flax varieties, the lack of high-quality genomes for different flax varieties remains an unresolved issue. The ideal genome sequence aims to achieve nearly complete chromosomal-level assembly, with gapless assembly of entire chromosomes [35]. ONT UL reads, Hi-C reads, and PacBio HiFi reads are all included into the assembly strategy. ONT UL reads were utilized to fill in any possible gaps in the genome, whereas PacBio HiFi reads served as the genomic backbone in this investigation. This strategy has also been applied to various crops; e.g. the genome assembly of *Citrus limon* uncovered genes linked to the biosynthesis of flavor compounds and the tolerance to *HLB* [36]; mulberry genome reveals new mechanism of centromere formation [37]. To better understand the evolutionary patterns and genetic differences among different flax varieties at the genomic level. Based on the oilseed and fiber variety Gaosi, we created a high-quality T2T flax genome. The results of this study not only enhance our understanding of the flax genome for oilseed and fiber varieties but also reveal numerous previously undiscovered regions within the genome. This is the first comprehensive study reporting the T2T genome of flax.

In this study, the oilseed and fiber variety Gaosi was used as the sequencing material, and a hybrid assembly strategy integrating sequencing data from multiple sources was employed to obtain the first complete and highest quality flax genome. The genome assembly size is 482.51 Mb, significantly larger than the previously reported flax genomes of Longya10 and CDC Bethune [30–32]. Furthermore, the genome assembled in this study has a contig N50 of 33.03 Mb, which is 17.03 times larger than the genomes of Longya10 (1.94 Mb) and CDC Bethune (0.023 Mb). When the two types of flax genomes were compared to the Longya10 genome, structural differences such as duplications, inversions, and translocations were found. These differences highlight the distinct genetic characteristics between oilseed flax varieties and dual-purpose oil-fiber flax varieties [31]. In the 15 chromosomes of flax, 30 telomeres and 15 centromeres were identified, which had not been reported previously. Regarding gene annotation, the Gaosi T2T genome contains 46 634 annotated genes, slightly more than the 43 484 genes in the CDC Bethune genome and the 43 500 genes in the Longya10 genome [31, 32]. In this study, the average length of CDS in the Gaosi T2T genome was 1151.92 bp, significantly longer than that of the Longya10 genome (238 bp). This discrepancy is probably due to improvements in assembly techniques and sequencing technologies [38]. The Gaosi T2T reference genome obtained in this study for the first time provides a theoretical foundation for breeding assisted by molecular markers and genetic studies functional studies in flax.

To gain a deeper understanding of flax evolution, we compared the evolutionary relationships among flax, species within the order Malpighiales, and several outgroup species. Previous studies have indicated that the genus flax diverged from the genus *Brassica* ~107.44 MYA [31]. However, results based on fossil calibration in this study indicate that flax diverged from the genus *Brassica* between 89.5 and 102.2 MYA. Previous research has also suggested that flax underwent a WGD event ~10 MYA [39]. In this study, it was discovered that flax underwent WGD events ~11.5, 53.5, and 114 MYA. Furthermore, flax experienced two distinct WGD events when the Ks values were ~0.15 and 0.7. These multiple WGD events have contributed to the presence of a large number of expanded genes in flax.

An essential phase in the production of plant oils is the stepwise desaturation of fatty acids, which establishes the proportion of unsaturated to saturated fatty acids [11]. The molecular processes that underlie the synthesis and storage of fatty acids in flax are yet unknown. The fatty acid metabolism pathway in flax was recreated in this work, and we discovered that the genes linked to it are found in numerous copies on either the same or distinct chromosomes. Most of these genes are significantly expressed in fruits 10–20 days after flowering, according to transcriptome research. According to earlier research, these multicopy genes show functional redundancy and operate in a variety of flax tissues [40]. This study also validated the concept of functional redundancy in fatty acid metabolic pathway genes through qRT-PCR experiments. However, the enzyme activities encoded by these genes should not be overlooked; when enzyme activity is high, excessive expression may not be necessary. Additionally, six new genes were annotated in the flax fatty acid metabolic pathway, offering fresh perspectives on the route. Further functional studies on these genes will help uncover the molecular mechanisms underlying flax fatty acid biosynthesis.

Rebuilding genomes with great continuity and completeness was made possible by the T2T assembly approach, which also offered new information about intricate repeating sequences. The most prevalent type of repeat in the flax genome was LTR retrotransposons, accounting for 20.3%, with LTR-Gypsy (6.75%) and LTR-Copia (4.21%) being the main subtypes. LTR transposons were significant repetitive sequences that had a considerable impact on many aspects of the genome. Studies had shown that transposon insertions into critical functional genes could lead to changes in certain traits, while insertions in other regions might regulate gene expression [41]. Fluctuations in the number of LTR transposons can result in alterations to the genome size. In *Pisum* species, the expression of the *PsMYB10.2* gene was able to be increased using LTR retrotransposons, thereby regulating the biosynthesis of anthocyanins [42]. Currently, studies on LTR retrotransposons in flax were limited. However, the identification of LTR transposons in the Gaosi T2T genome provided a starting point for additional research into the molecular processes behind important traits of LTRs in flax. The flax T2T genome’s annotated repetitive sequences were used to identify 40 transposons that overlapped with fatty acid biosynthesis-related genes. Studies have shown that LTRs could be differentially regulated under stress and preserved through natural selection, demonstrating adaptive potential and driving evolutionary processes [43]. These transposons can encourage the differentiation of gene functions and lead to the formation of novel characteristics in flax, providing new directions for research on gene function in its fatty acid metabolic pathway.

The *SAD* and *FAD* gene families encode essential fatty acid desaturases that play a role in the fatty acid biosynthesis pathway [9]. These enzymes were essential for plant metabolism because they maintained the integrity of cell membranes and made it easier for hormone signaling molecules to be synthesized [44]. In this study, homology-based techniques were used to identify 14 *FAD* genes and 4 *SAD* genes in flax, and these genes were classified into three subfamilies: *SAD*, *FAD3/FAD7/8*, and *FAD2*. According to gene structural analysis, the *SAD* and *FAD* genes have between 1 and 8 exons while having between 0 and 7 introns. Similar results in other plants have been documented, such as wheat and rapeseed, indicating that *SAD* and *FAD* constitute a highly conserved gene family [45, 46]. The promoter’s *cis*-acting elements are essential because they engage in and control gene expression. The majority of the components in the promoter regions of the *SAD* and *FAD* genes were discovered to be responsive to abiotic stressors (low-temperature and anaerobic conditions) and plant hormones (MeJA and abscisic acid). According to a study, the promoters of the sesame *SeFAD2* and rapeseed *BnFAD2A5–1* genes both contained ABRE and were made to express abscisic acid (ABA) [47]. In the stress response elements of the *SAD* and *FAD* genes, the plant’s low-temperature response was linked to LTR, whereas anaerobic induction was linked to ARE components [48, 49]. Thus, it is hypothesized that several transcription factors control the expression of the *SAD* and *FAD* genes, and their functions and mechanisms need further exploration.

Overall, we used UL sequencing data to successfully synthesize the first seamless T2T genome of flax. The assembly of the flax species genome was of milestone significance, establishing the groundwork for next studies in flax genomics and creating new opportunities to improve breeding practices, boost agricultural yields, and identify the genetic underpinning of species characteristics.

## Materials and methods

### Selection of plant materials and genome sequencing

This study focused on the oil and fiber dual-use flax variety (Gaosi) provided by the Chinese Heilongjiang Academy of Agricultural Sciences. After planting in the field for 30 days, flax plants produced vigorous, fresh leaves, and fruit tissues were collected at 10, 20, and 30 days after flowering, respectively. The samples were kept in a freezer set to −80°C and frozen in liquid nitrogen. Using the CTAB technique, genomic DNA (gDNA) was extracted from fresh leaves and then used for genome sequencing. The Trizol technique was used to extract total RNA from fruits. The SPARKscript II RT Plus Kit (With gDNA Eraser) (Shandong Sparkjade Biotechnology Co., Ltd.) was utilized to create cDNA, followed by quantitative real-time PCR (qPCR) performed with TransStart^®^ Top Green qPCR SuperMix.

To extract high-quality DNA from flax leaves, the PacBio library was built using the SMRTBell library approach. The PacBio Sequel II platform was used to do PacBio HiFi sequencing, and high-precision CCS reads were obtained after calibration. The Oxford Nanopore SQKLSK109 ligation kit was used to prepare a library for ONT UL sequencing, followed by sequencing on the Nanopore PromethION high-throughput sequencer. The Illumina HiSeq-2500 platform was used to prepare and sequence the Hi-C library. The TruSeq Nano DNA Library Prep Kit on the Illumina NovaSeq 6000 platform was used for both Illumina and RNA sequencing. Wuhan Beina Technology Co., Ltd. (Wuhan, China) supplied the sequencing work.

### Data filtering and genome assembly

Filtlong (v0.2.4) was used to filter ONT UL readings in order to exclude fragments <10 kb. Using CCS (v6.0.0) software, the quality of PacBio HiFi raw sequencing data was evaluated by removing reads with an SNR < 2.5 and data with fewer than three cycles. The program Fastp (v0.21.0) was used to filter second-generation data in order to eliminate the original reads [50]. Software called Fastp (v0.21.0) and HICUP (v0.8.0) were used to process Hi-C raw sequencing data, removing reads that could not be uniquely mapped, along with invalid pairs, self-circles, and dangling ends, to obtain valid data for further analysis.

NextDenovo (v2.5.0) was used to assemble the ONT UL sequencing data utilizing parameters (read_cutoff = 1 k, blocksize = 1 g, nextgraph_options = −a 1). Racon v1.4.11 software was used to apply two rounds of third-generation error correction to the preliminary assembly. The genome, corrected by third-generation sequencing, was further refined through two rounds of polishing using second-generation sequencing data with Pilon v1.23 software. PacBio HiFi data were assembled using two strategies: PacBio HiFi assembly (Hifiasm v0.16.1-r375) and PacBio HiFi + ONT UL mixed assembly (Hifiasm v0.18.2-r467) [51]. Mitochondrial and chloroplast genomes were aligned using minimap2 (2.17-r941), and bases with >50% alignment were removed. Bacterial contamination was eliminated by performing a BLAST search against the RefSeq database [52]. We comprehensively assessed the genome quality of each assembly version and ultimately selected the contig genome from the PacBio HiFi assembly strategy as the T2T genome scaffold. The T2T HiFi scaffold genome was then subjected to haplotype purging using the Purge_haplotigs software (v1.0.4).

ALLHIC (v0.9.8) software [53] was used to sequence and orient contigs within the n chromosome groups. Then, using 3D-DNA (v180419), the interactions between contigs were transformed into particular binary files [54] and Juicer (v1.6) software [55]. Based on this, contigs were manually sorted and oriented using Juicebox (v1.11.08) [56]. The sequenced, oriented, and deduplicated contig sequences were filled with 100 N to fill in the gaps, ultimately obtaining the genome sequence at the chromosome level. The intensity of the interactions between each chromosomal component was visualized using HiCExplorer v3.6. Afterwards, we performed telomere filling by first using Winnowmap (v1.11, parameter: k = 15, -MD) to align all sequences and collect all reads that were aligned once within 50 bp of the chromosome end [57].

The number of telomere repeat motifs (‘CCCTAA’/'TTTAGGG’) in each read was calculated, and the reference (ref) was the read with the highest count. Using Medaka_consensus’s (−m r941_min_high_g360) parameters, the telomere reads of the ref and other telomere reads were reassembled to obtain the consensus sequence. The telomere consensus sequences on each chromosome were compared using Nucmer (v3.1) [57], and the best alignment result was used to replace the telomere sequence. If the identity was <80% threshold, or if the alignment region was not within the terminal 20 kb of the chromosome, no replacement was made [58]. For gap filling in the genome, we used Winnowmap (v1.11) with the parameters (k = 15, –MD) to compare the gap-filling data (excluding N) with the genome gaps (including N). The data used for filling the gaps followed the priority order: other corrected genome versions > ONT data > PacBio HiFi data. If reads were present in the alignment results that spanned both ends of the replacement region, the gap filling for that region was considered reliable.

### Telomere and centromere identification

The telomeres of the majority of plants are made up of a number of extremely conserved microsatellites. Using VGP, the common plant telomere sequence (AAACCCT) was found on the flax’s 15 chromosomes. The telomere sequences were manually inspected and repaired using PacBio HiFi and ONT reads. The centromere prediction method was largely consistent with the identification methods used for T2T kiwifruit and myrtle [59, 60]. Tandem repeat sequences in the genome were detected using TRF software with the parameters (2 7 7 80 10 50 500 -d -h). Tandem repeat monomers on each chromosome were clustered using cd-hit with the parameters (−c 0.9 -n 5 -d 0 -g 1 -aS 0.9), where the corresponding tandem repeat sequence clusters were assigned to monomers that shared >90% of their similarities. The representative sequence from the largest tandem repeat sequence cluster was used as the library (lib). RepeatMasker v4.0.9 was then used with parameters (−nolow -no_is -norna -parallel 2 -lib trc_lib.fa) to search for the representative sequences across chromosomes. Finally, Bedtools merge was applied to integrate the centromeric regions for each chromosome. Bedtools was used with a 50-k window to calculate density, and the RIdeogram package was employed to visualize the centromeric regions.

### Annotation of the genome

RepeatModeler v1.0.11 was used to identify and classify repetitive sequences in the genome [61]. The new library was combined with the known repeat library, and the genome’s repeated sequences and transposon types were predicted using RepeatMasker v4.0.9. Next, the anticipated outcomes were deduplicated [62].

Gene structure prediction for the genome was carried out using transcriptome data, homology analysis, and *ab initio* sequence prediction. To determine the relevant coding frames, transcripts were rebuilt after raw readings were mapped to the genome. Augustus software was used for *de novo* prediction of the genomic regions covered by duplicated sequences [63]. Homology prediction was performed using the species *A. thaliana*, *L. tenue*, and *L. usitatissimum*. Gene sets from the three prediction techniques were combined using MAKER software. Open reading frames (ORFs) were predicted, validated, and deduplicated to generate a complete gene set [64]. Genome annotation completeness was assessed using BUSCO v5.2.2 (parameters: -m prot -c 40 –long -f). A BUSCO score >90% was considered to indicate good genome annotation quality [65]. The protein sequences and motifs were compared with several protein databases, including UniProt, Nr, GO, KOG, Pfam, InterPro, and KEGG, to verify their potential biological functions. After performing self-alignment of the protein sequences using Blastp, homologous segments were identified using MCScanX [66, 67]. Circos plots were generated using the CirclizeR package [68]. Further prediction of tRNAs was conducted using tRNAscan-SE v1.23 [69]. rRNAs were predicted using an rRNA database, while ncRNAs were identified based on INFERNAL v1.1.2 and the Pfam database [70].

### Genomic evolution analysis

To further investigate the link between flax’s biological evolution and functions, the genomes of 15 species were subjected to gene family grouping analysis (*V. vinifera*, *C. sativa*, *G. max*, *C. sativus, A. thaliana*, *B. sexangula*, *E. novogranatense*, *S. brachista*, *P. alba*, *H. brasiliensis*, *J. curcas*, *R. communis*, *E. peplus*, and *O. sativa*) using OrthoFinder v2.3.12 [71]. Using clusterProfiler, GO and KEGG functional annotation analysis was carried out on certain species’ gene families [72]. RaxML software (v8.2.10) was utilized to compute a maximum likelihood (ML) phylogenetic tree [73]. Species divergence times were computed using the PAML software’s mcmctree algorithm, based on the phylogenetic tree topology and fossil calibration points. Additionally, CAFE v3.1 was used for gene family contraction and expansion analysis [74]. The synonymous mutation frequency (Ks) and nonsynonymous mutation frequency (Ka) were computed using PAML v4.9’s yn00 module, and the Ka/Ks ratio to detect WGD events [75]. Using the formula T = Ks/2r × 10^−6^ to calculate the time for genome duplication, where r represents the constant nucleotide replacement rate (6.54 × 10^−9^). Gene density plots for species closely related to flax were generated using ggplot2 (v2.2.1), and synteny maps of these species’ genomes were created using JCVI (v0.9.13) [76].

### Finding the genes involved in fatty acid biosynthesis

Fatty acid biosynthesis genes in the flax T2T genome were identified by referencing the protein sequences of *Arabidopsis* fatty acid-related genes. Blastp was employed to query the T2T flax genome protein sequences using an e-value threshold of <10e^−50^. Gene filtering and refinement were conducted using SMART (http://smart.embl.de/smart/batch.pl) [66].

The transcriptome information was taken from flax flower tissues that were harvested 10, 20, and 30 days after flowering (PRJNA833557). The data were filtered using fastp [50], then aligned to the flax T2T genome. Expression quantification was performed using the R packages tidyverse, Rsubrad, limma, and edgeR. Finally, a heatmap of FPKM log2 values was generated using TBtools [77]. The primers specific to flax fatty acid-related genes are shown in Table S22. Transposable elements related to fatty acid biosynthesis pathways in flax were identified using the annotated repetitive sequence data from the T2T genome. Samples from Stages S1, S2, and S3 were pulverized into powder using a freeze dryer (SCIENTZ-18 N, Ningbo Scientz Biotechnology Co., Ltd., China). After that, they were combined with 1.0 ml of a 70% methanol solution and left to incubate overnight at 4°C. Following 10 min of centrifugation at 10 000 g, the mixture’s supernatant was gathered, filtered, and subjected to liquid chromatography–tandem mass spectrometry (LC–MS/MS) analysis (Q Exactive Orbitrap, Thermo Fisher Scientific, USA).

### Analysis of SAD and FAD gene families

The TAIR website provided the amino acid sequences for *Arabidopsis SAD* and *FAD* (https://www.arabidopsis.org/) [36]. In MEGA version 11, under default conditions, a phylogenetic tree was built using the maximum likelihood (ML) method on the *Arabidopsis* and flax SAD and FAD amino acid sequences (Neighbor-Joining; Bootstrap = 1000). Next, iTOL (https://itol.embl.de/) was used to visualize and improve the phylogenetic tree. The locations of the *SAD* and *FAD* genes on the chromosome were ascertained using the FASTA and GFF3 annotation files of the T2T Gaosi genome. The NCBI CD-search tool was used to find regions that fall under the conserved domain. The SAD and FAD amino acid sequences were subjected to motif analysis using MEME technology (http://alternate.meme-suite.org/tools/meme) [78], and TBtools was used to visualize the outcomes (version 2.069). Genomic nucleotide sequences 2000 bp upstream of all *SAD* and *FAD* genes were extracted using TBtools. The PlantCARE database was used to identify the *cis*-acting regulatory elements in the 2-kb upstream nucleotide sequence of the translation start site of the *FAD* and *SAD* genes.

## Supplementary Material

Web_Material_uhaf127

## Data Availability

The genome assembly data of the flax variety Gaosi has been archived at the China National Center for Bioinformation, with the project accession number PRJCA037526. All pertinent data related to this study are included in the supplementary materials that accompany this paper.
