# Supplementary material for: Telomere to telomere flax (Linum usitatissimum L.) genome assembly unlocks insights beyond fatty acid metabolism pathways
Source: Hortic Res. 2025 May 7;12(8):uhaf127. doi: 10.1093/hr/uhaf127 (PMC12265461; doi:10.1093/hr/uhaf127)
Supplement: Web_Material_uhaf127 [file web_material_uhaf127.zip › Supplementary Figures.docx]

**Supplementary Figures**


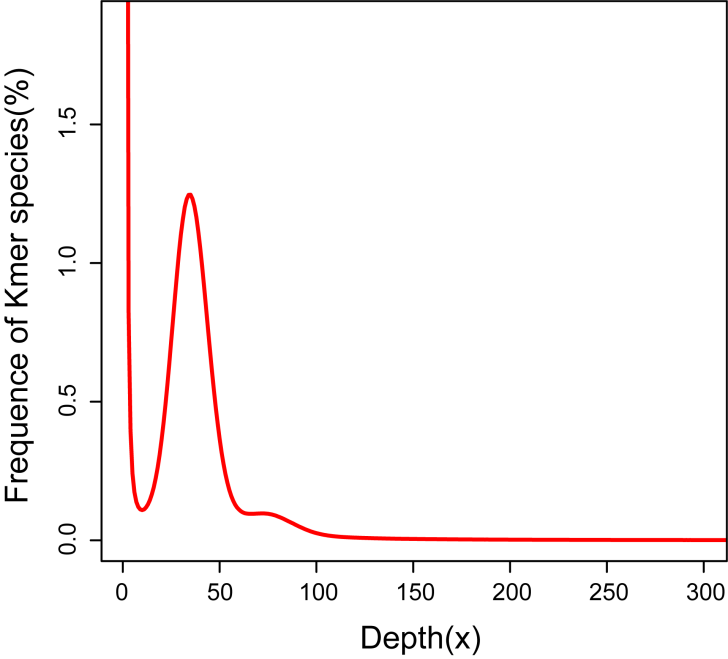


**Figure S1. K-mer depth-frequence distribution.**


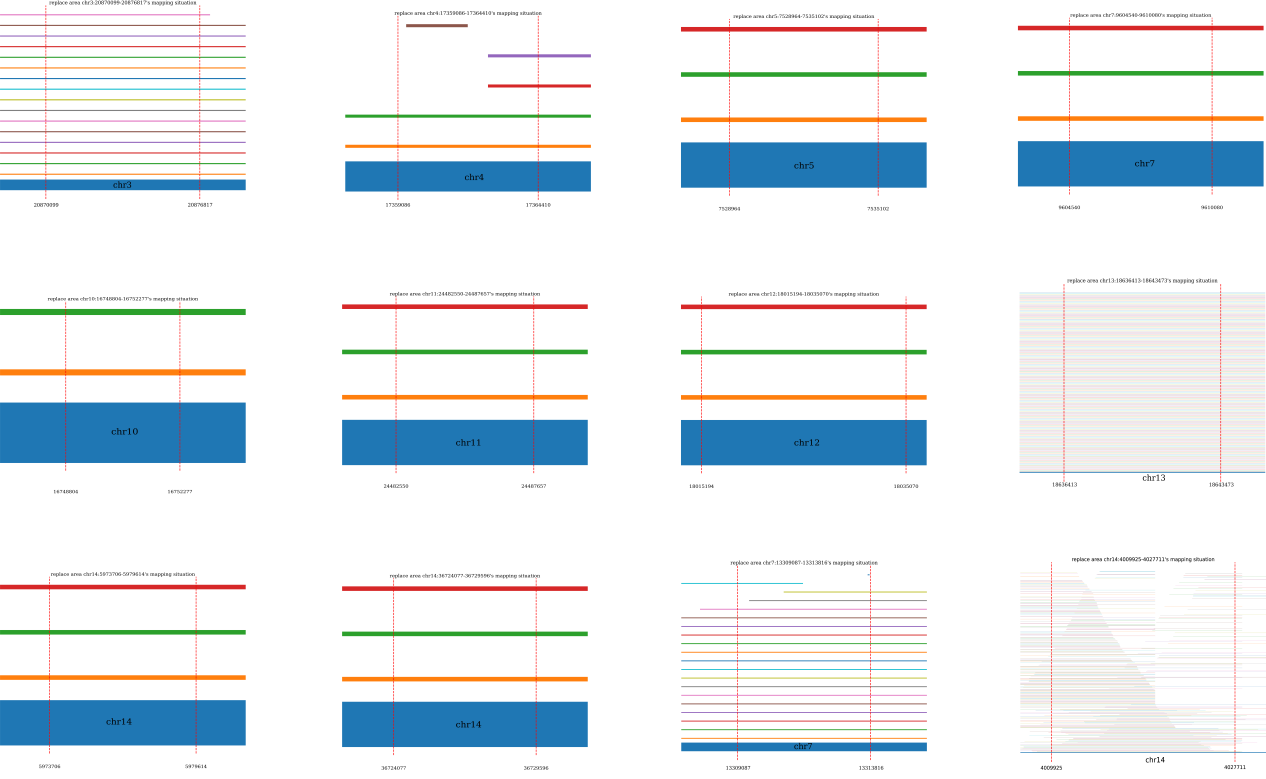


**Figure S2. Location information of gap filling in flax T2T genome.**


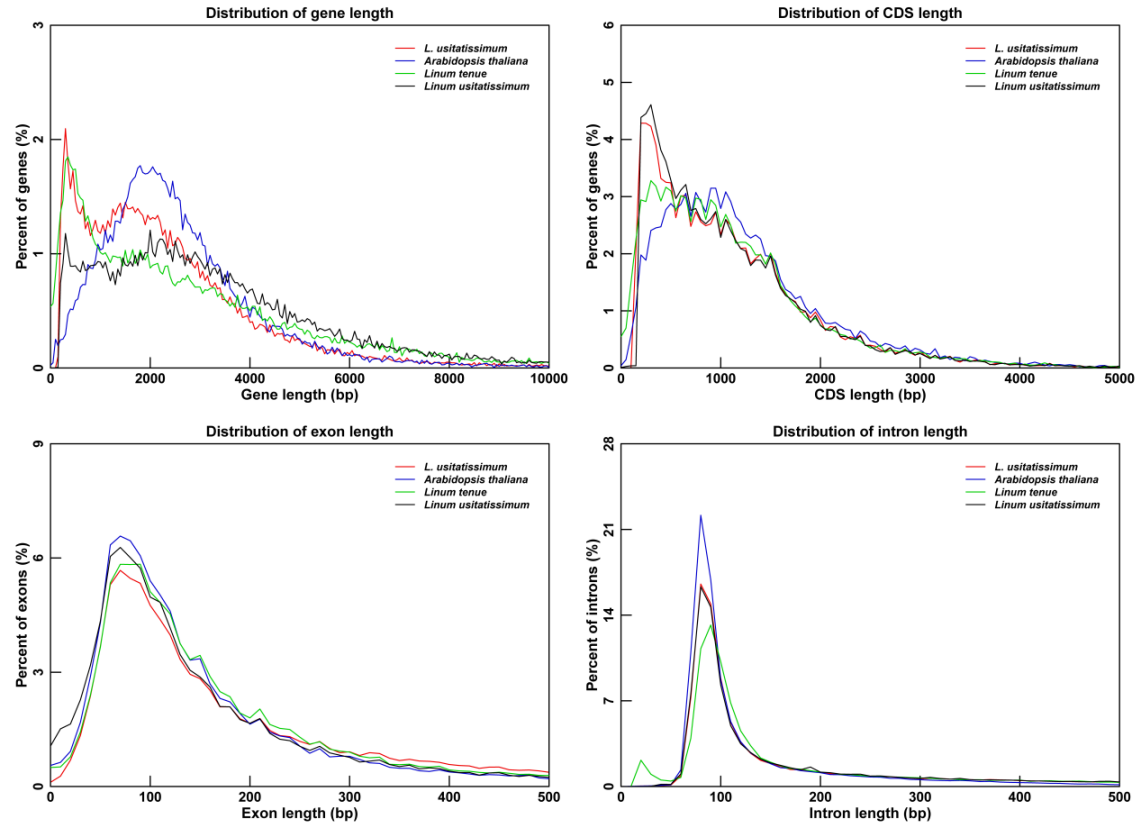


**Figure S3. Comparative study on gene length between flax and related species.**


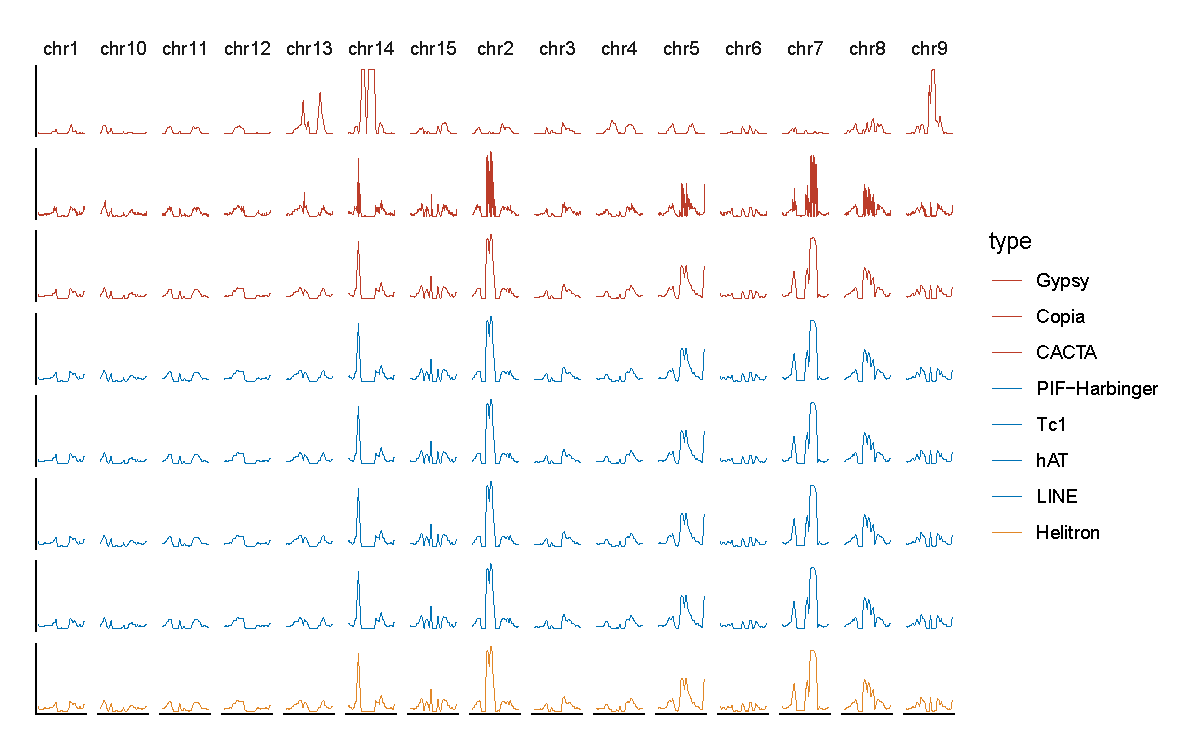


**Figure S4. The chromosome distribution of different types of TE in flax.**


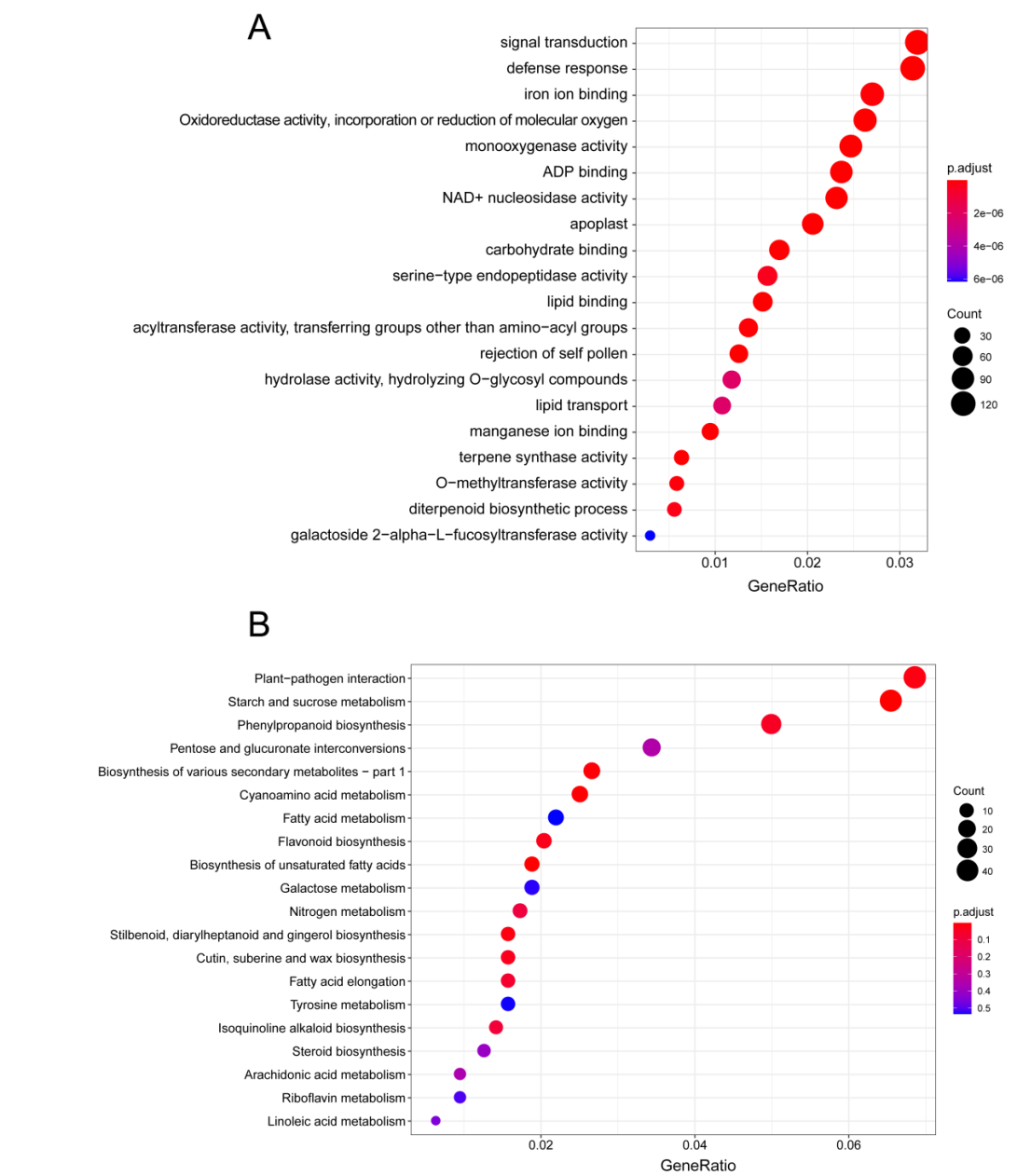


**Figure S5. GO and KEGG enrichment of unique gene families in flax.**


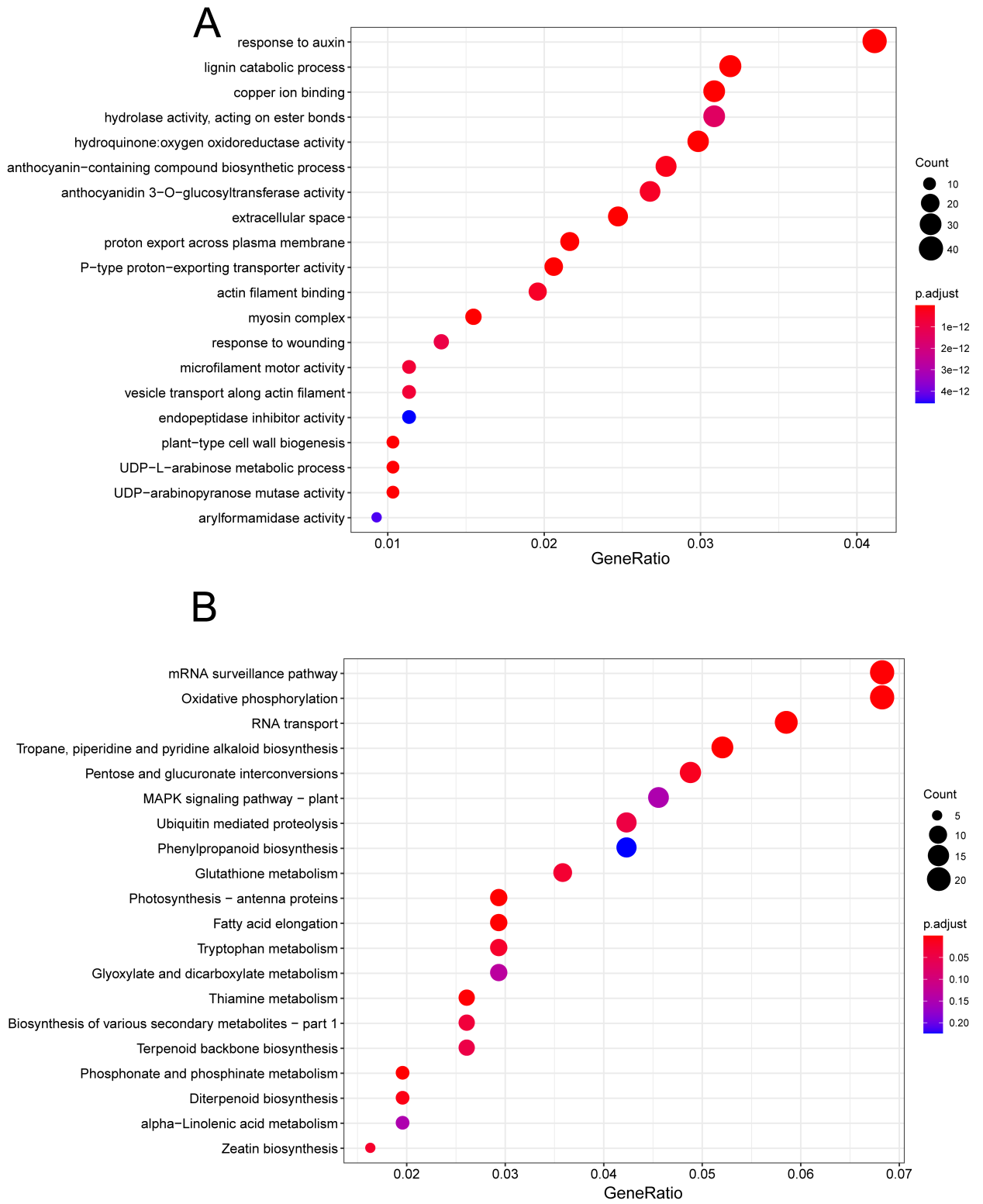


**Figure S6. Enrichment analysis of flax expansion genes KEGG and GO. (A) GO enrichment analysis of expanded genes (B) KEGG enrichment analysis of amplified genes.**


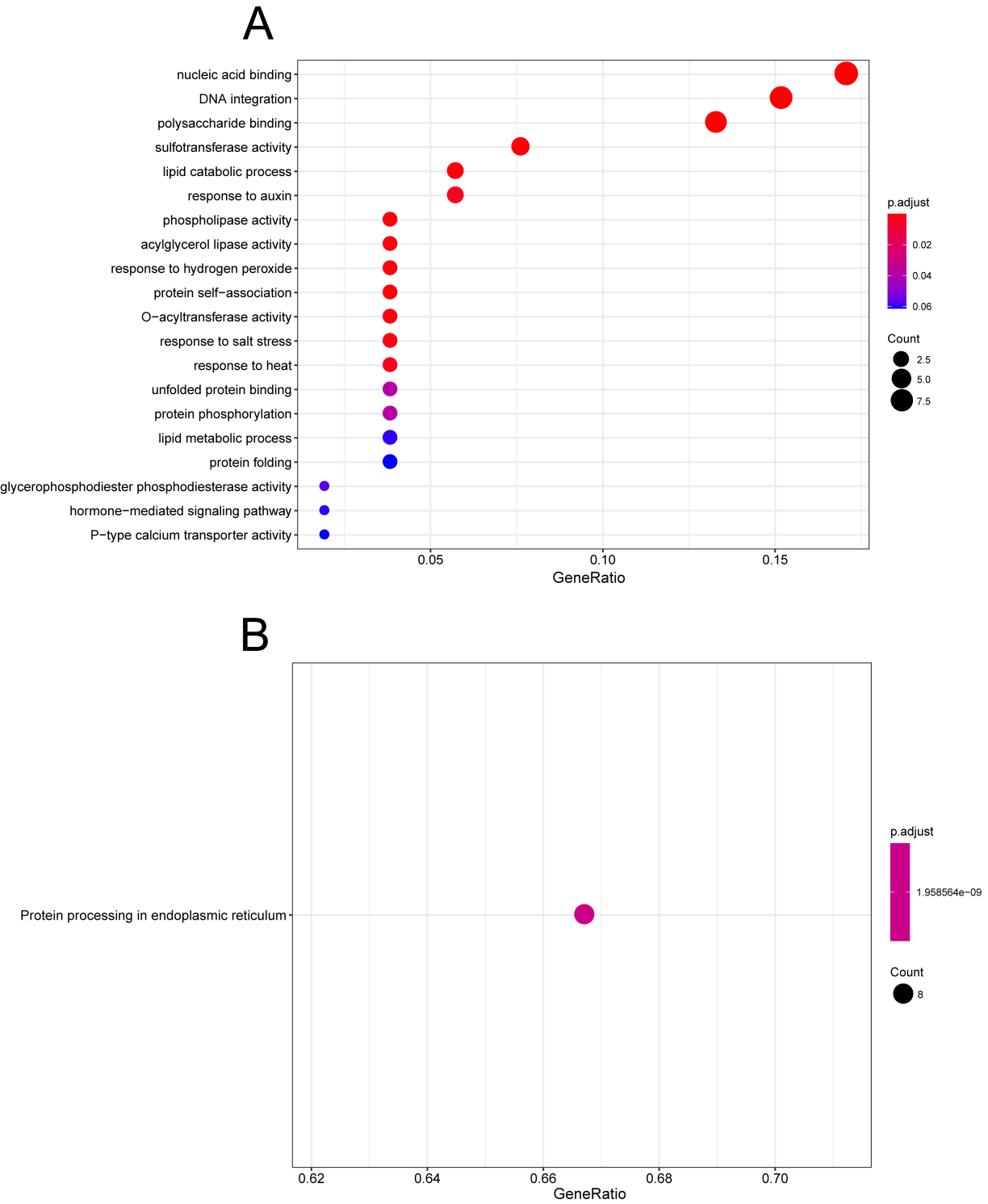


**Figure S7. Enrichment analysis of flax contraction genes KEGG and GO. (A) GO enrichment analysis of contraction genes (B) KEGG enrichment analysis of contraction genes.**
