# Supplementary material for: Telomere to telomere flax (Linum usitatissimum L.) genome assembly unlocks insights beyond fatty acid metabolism pathways
Source: Hortic Res. 2025 May 7;12(8):uhaf127. doi: 10.1093/hr/uhaf127 (PMC12265461; doi:10.1093/hr/uhaf127)
Supplement: Web_Material_uhaf127 [file web_material_uhaf127.zip › Supplementary Tables.docx]

**Table S1. Statistical analysis of K-mer.**

| Parameter | Value |
| --- | --- |
| K-mer | 19 |
| K-mer Number | 16,732,527,498 |
| K-mer Depth | 36.40 |
| Predicting genome Size (Mb) | 460.13 |
| Data Size(Gb) | 20.86 |
| Depth | 45.34 |
| Heterozygous Ratio(%) | 0.34 |
| Duplication Ratio(%) | 55.62 |

**Table S2. Statistics on ONT Ultra long sequencing data volume for the third generation.**

| Rank | Flag | Total Base | TotalReads | MaxLen | AvgLen | N50 | L50 | N90 | L90 | MeanQ |
| --- | --- | --- | --- | --- | --- | --- | --- | --- | --- | --- |
| >0 | all | 16,685,900,590 | 162,237 | 1,119,227 | 102,848.92 | 100,000 | 68,859 | 84,170 | 142,037 | 10.42 |
| >0 | pass | 15,736,918,200 | 153,062 | 393,662 | 102,814.01 | 100,000 | 64,969 | 84,121 | 134,000 | 10.71 |
| >0 | fail | 948,982,390 | 9,175 | 1,119,227 | 103,431.32 | 100,002 | 3,890 | 84,917 | 8,039 | 5.60 |
| >10000 | all | 16,685,900,590 | 162,237 | 1,119,227 | 102,848.92 | 100,000 | 68,859 | 84,170 | 142,037 | 10.42 |
| >10000 | pass | 15,736,918,200 | 153,062 | 393,662 | 102,814.01 | 100,000 | 64,969 | 84,121 | 134,000 | 10.71 |
| >10000 | fail | 948,982,390 | 9,175 | 1,119,227 | 103,431.32 | 100,002 | 3,890 | 84,917 | 8,039 | 5.60 |
| >20000 | all | 16,685,900,590 | 162,237 | 1,119,227 | 102,848.92 | 100,000 | 68,859 | 84,170 | 142,037 | 10.42 |
| >20000 | pass | 15,736,918,200 | 153,062 | 393,662 | 102,814.01 | 100,000 | 64,969 | 84,121 | 134,000 | 10.71 |
| >20000 | fail | 948,982,390 | 9,175 | 1,119,227 | 103,431.32 | 100,002 | 3,890 | 84,917 | 8,039 | 5.60 |
| >30000 | all | 16,685,900,590 | 162,237 | 1,119,227 | 102,848.92 | 100,000 | 68,859 | 84,170 | 142,037 | 10.42 |
| >30000 | pass | 15,736,918,200 | 153,062 | 393,662 | 102,814.01 | 100,000 | 64,969 | 84,121 | 134,000 | 10.71 |
| >30000 | fail | 948,982,390 | 9,175 | 1,119,227 | 103,431.32 | 100,002 | 3,890 | 84,917 | 8,039 | 5.60 |
| >40000 | all | 16,685,900,590 | 162,237 | 1,119,227 | 102,848.92 | 100,000 | 68,859 | 84,170 | 142,037 | 10.42 |
| >40000 | pass | 15,736,918,200 | 153,062 | 393,662 | 102,814.01 | 100,000 | 64,969 | 84,121 | 134,000 | 10.71 |
| >40000 | fail | 948,982,390 | 9,175 | 1,119,227 | 103,431.32 | 100,002 | 3,890 | 84,917 | 8,039 | 5.60 |
| >50000 | all | 16,685,900,590 | 162,237 | 1,119,227 | 102,848.92 | 100,000 | 68,859 | 84,170 | 142,037 | 10.42 |
| >50000 | pass | 15,736,918,200 | 153,062 | 393,662 | 102,814.01 | 100,000 | 64,969 | 84,121 | 134,000 | 10.71 |
| >50000 | fail | 948,982,390 | 9,175 | 1,119,227 | 103,431.32 | 100,002 | 3,890 | 84,917 | 8,039 | 5.60 |
| >60000 | all | 16,685,900,590 | 162,237 | 1,119,227 | 102,848.92 | 100,000 | 68,859 | 84,170 | 142,037 | 10.42 |
| >60000 | pass | 15,736,918,200 | 153,062 | 393,662 | 102,814.01 | 100,000 | 64,969 | 84,121 | 134,000 | 10.71 |
| >60000 | fail | 948,982,390 | 9,175 | 1,119,227 | 103,431.32 | 100,002 | 3,890 | 84,917 | 8,039 | 5.60 |
| >70000 | all | 16,685,900,590 | 162,237 | 1,119,227 | 102,848.92 | 100,000 | 68,859 | 84,170 | 142,037 | 10.42 |
| >70000 | pass | 15,736,918,200 | 153,062 | 393,662 | 102,814.01 | 100,000 | 64,969 | 84,121 | 134,000 | 10.71 |
| >70000 | fail | 948,982,390 | 9,175 | 1,119,227 | 103,431.32 | 100,002 | 3,890 | 84,917 | 8,039 | 5.60 |
| >80000 | all | 16,685,900,590 | 162,237 | 1,119,227 | 102,848.92 | 100,000 | 68,859 | 84,170 | 142,037 | 10.42 |
| >80000 | pass | 15,736,918,200 | 153,062 | 393,662 | 102,814.01 | 100,000 | 64,969 | 84,121 | 134,000 | 10.71 |
| >80000 | fail | 948,982,390 | 9,175 | 1,119,227 | 103,431.32 | 100,002 | 3,890 | 84,917 | 8,039 | 5.60 |
| >90000 | all | 12,167,663,999 | 109,269 | 1,119,227 | 111,355.13 | 108,027 | 47,087 | 92,878 | 95,959 | 10.41 |
| >90000 | pass | 11,469,425,743 | 103,015 | 393,662 | 111,337.43 | 108,040 | 44,409 | 92,879 | 90,468 | 10.70 |
| >90000 | fail | 698,238,256 | 6,254 | 1,119,227 | 111,646.67 | 107,788 | 2,679 | 92,862 | 5,491 | 5.62 |
| >100000 | all | 8,342,911,928 | 68,858 | 1,119,227 | 121,161.11 | 117,426 | 30,073 | 102,703 | 60,624 | 10.41 |
| >100000 | pass | 7,868,392,450 | 64,968 | 393,662 | 121,111.82 | 117,426 | 28,391 | 102,731 | 57,203 | 10.69 |
| >100000 | fail | 474,519,478 | 3,890 | 1,119,227 | 121,984.44 | 117,408 | 1,682 | 102,375 | 3,422 | 5.64 |
| >110000 | all | 5,631,017,940 | 42,932 | 1,119,227 | 131,161.32 | 127,099 | 18,948 | 112,608 | 37,873 | 10.40 |
| >110000 | pass | 5,310,557,235 | 40,517 | 393,662 | 131,069.85 | 127,092 | 17,897 | 112,606 | 35,746 | 10.69 |
| >110000 | fail | 320,460,705 | 2,415 | 1,119,227 | 132,695.94 | 127,250 | 1,051 | 112,624 | 2,128 | 5.65 |
| >120000 | all | 3,761,794,528 | 26,621 | 1,119,227 | 141,309.29 | 137,000 | 11,851 | 122,533 | 23,519 | 10.40 |
| >120000 | pass | 3,547,789,967 | 25,135 | 393,662 | 141,149.39 | 136,988 | 11,203 | 122,514 | 22,209 | 10.68 |
| >120000 | fail | 214,004,561 | 1,486 | 1,119,227 | 144,013.84 | 137,404 | 648 | 122,831 | 1,310 | 5.65 |
| >130000 | all | 2,505,815,440 | 16,538 | 1,119,227 | 151,518.65 | 146,835 | 7,415 | 132,570 | 14,630 | 10.38 |
| >130000 | pass | 2,360,051,289 | 15,600 | 393,662 | 151,285.34 | 146,825 | 7,008 | 132,567 | 13,803 | 10.67 |
| >130000 | fail | 145,764,151 | 938 | 1,119,227 | 155,398.88 | 147,006 | 408 | 132,663 | 827 | 5.66 |
| >140000 | all | 1,667,391,705 | 10,308 | 1,119,227 | 161,757.05 | 156,667 | 4,646 | 142,536 | 9,128 | 10.37 |
| >140000 | pass | 1,569,902,223 | 9,729 | 393,662 | 161,363.16 | 156,541 | 4,397 | 142,535 | 8,618 | 10.65 |
| >140000 | fail | 97,489,482 | 579 | 1,119,227 | 168,375.62 | 158,844 | 250 | 142,566 | 511 | 5.64 |
| >150000 | all | 1,099,906,639 | 6,384 | 1,119,227 | 172,291.14 | 167,028 | 2,889 | 152,608 | 5,657 | 10.34 |
| >150000 | pass | 1,033,904,197 | 6,023 | 393,662 | 171,659.34 | 166,729 | 2,736 | 152,560 | 5,340 | 10.62 |
| >150000 | fail | 66,002,442 | 361 | 1,119,227 | 182,832.25 | 171,692 | 154 | 153,810 | 318 | 5.65 |
| >160000 | all | 728,766,908 | 3,983 | 1,119,227 | 182,969.35 | 176,996 | 1,808 | 162,653 | 3,532 | 10.28 |
| >160000 | pass | 682,463,471 | 3,749 | 393,662 | 182,038.8 | 176,673 | 1,711 | 162,620 | 3,326 | 10.57 |
| >160000 | fail | 46,303,437 | 234 | 1,119,227 | 197,877.94 | 183,333 | 98 | 163,409 | 206 | 5.60 |
| >170000 | all | 489,776,547 | 2,531 | 1,119,227 | 193,511.08 | 187,782 | 1,151 | 172,655 | 2,246 | 10.22 |
| >170000 | pass | 454,836,274 | 2,366 | 393,662 | 192,238.49 | 187,322 | 1,083 | 172,639 | 2,101 | 10.54 |
| >170000 | fail | 34,940,273 | 165 | 1,119,227 | 211,759.23 | 197,528 | 68 | 172,840 | 145 | 5.63 |
| >180000 | all | 324,792,351 | 1,586 | 1,119,227 | 204,787.11 | 198,227 | 722 | 182,543 | 1,407 | 10.13 |
| >180000 | pass | 299,813,178 | 1,478 | 393,662 | 202,850.59 | 197,365 | 679 | 182,442 | 1,313 | 10.47 |
| >180000 | fail | 24,979,173 | 108 | 1,119,227 | 231,288.64 | 208,322 | 44 | 184,032 | 95 | 5.50 |
| >190000 | all | 222,327,501 | 1,031 | 1,119,227 | 215,642.58 | 208,554 | 470 | 192,736 | 915 | 9.96 |
| >190000 | pass | 202,518,067 | 951 | 393,662 | 212,952.75 | 208,033 | 439 | 192,354 | 846 | 10.35 |
| >190000 | fail | 19,809,434 | 80 | 1,119,227 | 247,617.92 | 218,373 | 32 | 196,324 | 70 | 5.39 |
| >200000 | all | 153,103,368 | 675 | 1,119,227 | 226,819.8 | 218,373 | 307 | 203,027 | 600 | 9.86 |
| >200000 | pass | 137,607,873 | 617 | 393,662 | 223,027.35 | 217,507 | 286 | 202,882 | 549 | 10.30 |
| >200000 | fail | 15,495,495 | 58 | 1,119,227 | 267,163.71 | 232,015 | 22 | 204,890 | 51 | 5.22 |

**Table S3. Sequencing data statistics for PacBio HiFi.**

| Parameter | Value |
| --- | --- |
| HiFi Reads num | 1,356,275 |
| Total Bases(Gb) | 21.95 |
| HiFi Read length(bp) | 16,190.2 |
| HIFI reads length N50(bp) | 16,050 |

**Table S4. Sequencing data statistics for next-generation.**

| Parameter | Raw data | Clean data |
| --- | --- | --- |
| Number of reads | 143,795,716 | 143,751,096 |
| Number of bases | 21,569,357,400 | 20,860,750,881 |
| GC content (%) | 38.88 | 38.47 |
| Q20(%) | 98.15 | 98.16 |
| Q30(%) | 93.90 | 93.91 |

**Table S5. Hi-C sequencing data statistics.**

| Parameter | Value |
| --- | --- |
| Raw reads | 375,981,538 |
| Raw bases | 56,397,230,700 |
| Clean reads | 375,981,538 |
| Clean bases | 55,906,217,494 |
| Q20 rate(%) | 98.723 |
| Q30 rate(%) | 94.978 |

**Table S6. Genome evaluation table at contig level for three assembly strategies.**

| Parameter | Hifiasm(hifi+ont) | Hifiasm(hifi) | Nextdenovo(ont) |
| --- | --- | --- | --- |
| Genome size(bp) | 516,625,388 | 484,534,409 | 338,681,471 |
| Contig N50(bp) | 31,839,025 | 24,460,963 | 10,096,899 |
| Contig number | 284 | 61 | 99 |
| QV | 46.19 | 48.51 | 31.73 |
| Busco(%) | 97.1 | 97.2 | 97.5 |

**Table S7 Gap statistics of flax T2T genome.**

| Gap ID | Position | Start | End | Length(bp) |
| --- | --- | --- | --- | --- |
| Gap 1 | chr3 | 20870099 | 20876817 | 6718 |
| Gap 2 | chr4 | 17359086 | 17364410 | 5324 |
| Gap 3 | chr5 | 7528964 | 7535102 | 6138 |
| Gap 4 | chr7 | 9604540 | 9610080 | 5107 |
| Gap 5 | chr7 | 13309087 | 13313816 | 5540 |
| Gap 6 | chr10 | 16748804 | 16752277 | 3473 |
| Gap 7 | chr11 | 24482550 | 24487657 | 7060 |
| Gap 8 | chr12 | 18015194 | 18035070 | 5908 |
| Gap 9 | chr13 | 18636413 | 18643473 | 5519 |
| Gap 10 | chr14 | 4009925 | 4027711 | 4729 |
| Gap 11 | chr14 | 5973706 | 5979614 | 19876 |
| Gap 12 | chr14 | 36724077 | 36729596 | 17786 |

**Table S8. Genome consistency assessment.**

| Parameter | Ultra-long ONT | Hifi | Nextdenovo |
| --- | --- | --- | --- |
| Mapping rate(%) | 100 | 99.98 | 99.71 |
| Average sequencing depth | 31.81 | 45.31 | 42.3 |
| Coverage(%) | 99.96 | 99.86 | 99.81 |
| Coverage at least 4X(%) | 99.89 | 99.8 | 99.44 |
| Coverage at least 10X(%) | 99.77 | 99.75 | 98.73 |
| Coverage at least 20X(%) | 95.1 | 99.01 | 95.45 |

**Table S9. BUSCO evaluation statistics.**

| Parameter | Value | Percent(%) |
| --- | --- | --- |
| Complete BUSCOs (C) | 1570 | 97.3 |
| Complete and single-copy BUSCOs (S) | 463 | 28.7 |
| Complete and duplicated BUSCOs (D) | 1107 | 68.6 |
| Fragmented BUSCOs (F) | 7 | 0.4 |
| Missing BUSCOs (M) | 37 | 2.3 |
| Total BUSCO groups searched | 1614 | 100 |

**Table S10. Statistical analysis of QV values of various chromosomes in the flax genome.**

| Chromosome | QV |
| --- | --- |
| chr1 | 48.0012 |
| chr2 | 48.7461 |
| chr3 | 47.6268 |
| chr4 | 47.8652 |
| chr5 | 47.9799 |
| chr6 | 45.1088 |
| chr7 | 47.004 |
| chr8 | 48.2566 |
| chr9 | 46.3617 |
| chr10 | 49.0143 |
| chr11 | 48.6114 |
| chr12 | 47.0352 |
| chr13 | 47.9168 |
| chr14 | 46.3787 |
| chr15 | 46.8908 |

**Table S11. Basic statistics for predicting the gene structure of flax T2T genome.**

| Method | Software | Species | Gene number | Average gene length (bp) | Average CDS length (bp) | Average exon per gene | Average exon length (bp) | Average intron length (bp) |
| --- | --- | --- | --- | --- | --- | --- | --- | --- |
| Ab initio | GlimmmerHMM |  | 62,175 | 2,023.09 | 911.43 | 3.71 | 245.89 | 410.72 |
|  | AUGUSTUS |  | 48,081 | 2,394.77 | 1,190.53 | 5.18 | 230.03 | 288.4 |
| Homology | Exonerate | Arabidopsis thaliana | 50,961.00 | 4,315.94 | 912.23 | 4.2 | 217.39 | 1,064.88 |
|  |  | Linum tenue | 63,160 | 5,194.38 | 993.25 | 4.03 | 246.58 | 1,387.36 |
|  |  | Linum usitatissimum | 130,846 | 7,132.31 | 836.79 | 3.92 | 213.45 | 2,155.76 |
| RNAseq | TransDecoder |  | 43,971 | 2,935.27 | 964.65 | 5.27 | 357.34 | 246.78 |
| Integration | EVM |  | 45,874 | 2,307.69 | 1,176.55 | 5.14 | 228.93 | 273.26 |
| Final set | Anno-self |  | 46,634 | 2,531.75 | 1,151.92 | 5.14 | 280.53 | 263.78 |

**Table S12 . Functional annotation of genes encoding the T2T genome of flax.**

| Item | Count | Percentage |
| --- | --- | --- |
| Annotation | 42,805 | 91.79% |
| KEGG | 17,460 | 37.44% |
| Pathway | 8,943 | 19.18% |
| Nr | 39,864 | 85.48% |
| Uniprot | 39,824 | 85.40% |
| GO | 29,975 | 64.28% |
| KOG | 3,890 | 8.34% |
| Pfam | 31,531 | 67.61% |
| Interpro | 42,153 | 90.39% |

**Table S13 . Statistics of T2T genome repeat sequences in flax.**

|  | TE protiens | | De novo + repbase | | Combined TEs | |
| --- | --- | --- | --- | --- | --- | --- |
| Type | Length (bp) | % in genome | Length (bp) | % in genome | Length (bp) | % in genome |
| DNA | 2,512,015 | 0.52 | 121,479,807 | 25.18 | 122,174,743 | 25.32 |
| LINE | 3,340,874 | 0.69 | 41,783,634 | 8.66 | 42,664,876 | 8.84 |
| SINE | 0 | 0 | 743,561 | 0.15 | 743,561 | 0.15 |
| LTR | 32,240,498 | 6.68 | 96,214,812 | 19.94 | 97,945,348 | 20.3 |
| LTR-Gypsy | 19,053,529 | 3.95 | 31,362,210 | 6.5 | 32,549,796 | 6.75 |
| LTR-Copia | 13,129,231 | 2.72 | 19,524,518 | 4.05 | 20,307,591 | 4.21 |
| Other | 762 | 0 | 615 | 0 | 1,377 | 0 |
| Unknown | 41,052 | 0.01 | 40,905,202 | 8.48 | 40,946,112 | 8.49 |
| Total | 37,846,466 | 7.84 | 286,868,493 | 59.45 | 289,722,359 | 60.05 |

**Table S14. Basic statistics of T2T genome repeat sequences in flax.**

| Type |  | Copy | Average length(bp) | Total length(bp) | % of genome |
| --- | --- | --- | --- | --- | --- |
| miRNA |  | 170 | 122 | 20699 | 0.0043 |
| tRNA |  | 1255 | 75 | 93986 | 0.0195 |
| rRNA | rRNA | 23130 | 141 | 3252351 | 0.6741 |
|  | 18S | 9 | 2037 | 18335 | 0.0038 |
|  | 28S | 125 | 4041 | 505179 | 0.1047 |
|  | 5.8S | 1406 | 116 | 163083 | 0.0338 |
|  | 5S | 21590 | 119 | 2565754 | 0.5318 |
| snRNA | snRNA | 759 | 121 | 92108 | 0.0191 |
|  | CD-box | 463 | 111 | 51414 | 0.0107 |
|  | HACA-box | 80 | 128 | 10213 | 0.0021 |
|  | splicing | 216 | 141 | 30481 | 0.0063 |
|  | scaRNA | 0 | 0 | 0 | 0 |

**Table S15. Telomeres in the T2T genome of flax.**

| Chromosome | Length(bp) | Upstream Repeat number | Upstream position range (bp) | Downstream Repeat number | DownStream position range (bp) |
| --- | --- | --- | --- | --- | --- |
| chr1 | 33,029,367 | 2489 | 1-19345 | 1159 | 33009784l-33029154 |
| chr2 | 35,970,916 | 1777 | 14-12919 | 1317 | 35961163-35970913 |
| chr3 | 29,256,300 | 1458 | 4-17128 | 1423 | 29243440-29256298 |
| chr4 | 29,895,889 | 1378 | 8-18585 | 1337 | 29885916-29895877 |
| chr5 | 28,061,050 | 2482 | 1-20000 | 2622 | 28041054-28061050 |
| chr6 | 31,949,314 | 1765 | 1-19973 | 2700 | 31929317-31949314 |
| chr7 | 31,944,761 | 1668 | 1-20000 | 2767 | 31924766-31944761 |
| chr8 | 36,541,058 | 1579 | 19-12160 | 2282 | 36521059-36541058 |
| chr9 | 34,236,859 | 1911 | 1-19993 | 2745 | 34216864-34236859 |
| chr10 | 33,576,016 | 1410 | 109-16714 | 2671 | 33556023-33576016 |
| chr11 | 31,684,038 | 2467 | 1-19994 | 1169 | 31674620-31684035 |
| chr12 | 24,812,737 | 1730 | 1-12757 | 2771 | 24792744-24812737 |
| chr13 | 27,425,342 | 1045 | 4-16993 | 2785 | 27405348-27425342 |
| chr14 | 40,705,596 | 2441 | 1-19996 | 2574 | 40685603-40705596 |
| chr15 | 33,415,654 | 2344 | 1-20000 | 2500 | 33395660-33415654 |

**Table S16. Statistics of centromere repeat sequences in the T2T genome of flax.**

| Chromosome | Length(bp) | CE_start (bp) | CE_end (bp) |
| --- | --- | --- | --- |
| chr1 | 33029367 | 13935437 | 16011396 |
| chr2 | 35970916 | 11052026 | 16535083 |
| chr3 | 29256300 | 8704823 | 11664719 |
| chr4 | 29895889 | 13403594 | 15355821 |
| chr5 | 28061050 | 10352688 | 13812812 |
| chr6 | 31949314 | 11289735 | 15195801 |
| chr7 | 31944761 | 23866119 | 26892594 |
| chr8 | 36541058 | 10458560 | 14157552 |
| chr9 | 34236859 | 14209071 | 17248263 |
| chr10 | 33576016 | 13739779 | 17341756 |
| chr11 | 31684038 | 8796277 | 12487416 |
| chr12 | 24812737 | 11165390 | 19582040 |
| chr13 | 27425342 | 13773918 | 18241282 |
| chr14 | 40705596 | 27241922 | 27378399 |
| chr15 | 33415654 | 16479511 | 20075950 |

Note: CE_start indicates the centromeres start position upstream of the chromosome; CE_end is the termination site of centromeres downstream of the chromosome

**Table S17. Statistical analysis of gene family clustering results.**

| Species | Genes number | Genes in families | Family number | Unique families | Average genes per family |
| --- | --- | --- | --- | --- | --- |
| A.thaliana | 27557 | 27557 | 15725 | 2999 | 1.75 |
| B.sexangula | 26038 | 26038 | 16626 | 3407 | 1.57 |
| C.sativa | 25293 | 19497 | 13184 | 595 | 1.48 |
| C.sativus | 19497 | 19497 | 13184 | 595 | 1.48 |
| E.novogranatense | 28604 | 28604 | 15583 | 1865 | 1.84 |
| E.peplus | 25473 | 25473 | 15726 | 1911 | 1.62 |
| G.max | 47059 | 47059 | 16703 | 3121 | 2.82 |
| H.brasiliensis | 36703 | 36703 | 15573 | 1231 | 2.36 |
| J.curcas | 19419 | 19419 | 13534 | 395 | 1.43 |
| L.usitatissimum | 46634 | 46634 | 20286 | 7103 | 2.3 |
| O.sativa | 28735 | 28735 | 15982 | 4052 | 1.8 |
| P.alba | 30629 | 30629 | 14751 | 754 | 2.08 |
| R.communis | 20511 | 20511 | 14261 | 256 | 1.44 |
| S.brachista | 30209 | 30209 | 16262 | 2622 | 1.86 |
| V.vinifera | 25182 | 25182 | 14750 | 974 | 1.71 |

**Table S18. Fatty acid content of fruit tissue in three periods.**

| Compound Name | L10-1 content | L10-2 content | L10-3 content | L20-1 content | L20-2 content | L20-3 content | L30-1 content | L30-2 content | L30-3 content |
| --- | --- | --- | --- | --- | --- | --- | --- | --- | --- |
| palmitic acid | 301.04 | 252.97 | 293.83 | 310.25 | 296.51 | 221.78 | 350.03 | 182.27 | 258.57 |
| linoleic acid | 368.30 | 335.80 | 341.43 | 344.36 | 420.64 | 343.79 | 264.24 | 209.80 | 274.74 |
| linolenic acid | 803.15 | 688.32 | 778.78 | 1555.81 | 1571.76 | 1351.78 | 2083.50 | 1710.87 | 1770.19 |
| oleic acid | 595.60 | 478.81 | 557.20 | 1150.06 | 1153.60 | 886.37 | 1311.19 | 1010.72 | 928.74 |
| stearic acid | 135.64 | 110.70 | 124.11 | 239.24 | 222.70 | 169.66 | 344.97 | 263.23 | 261.65 |

**Table S19. Gene mining of flax fatty acid metabolism pathway.**

| Gene | Gene ID in Gaosi_T2T Genome | Chromosome Location  (bp) | CDS  Length | Gene ID in Longya10 Genome | Pfam database annotation |
| --- | --- | --- | --- | --- | --- |
| AACase | LusiChr9G109570.1 | chr9:24079771:24082633 | 1122 | L.us.o.m.scaffold12.69 | PF01039.25: Carboxyl_transferase domain |
| MCAT | LusiChr3G356900.1 | chr3:3526110:3529507 | 1191 | L.us.o.m.scaffold96.77 | PF00698.24: Acyl transferase domain |
|  | LusiChr1G193830.1 | chr1:3455643:3458429 | 1191 | L.us.o.m.scaffold139.165 |  |
| KAS III | LusiChr13G411420.1 | chr13:1200701:1203724 | 1209 | L.us.o.m.scaffold76.260 | PF08545.13: 3-Oxoacyl-[acyl-carrier-protein (ACP)] synthase III  PF08541.13: 3-Oxoacyl-[acyl-carrier-protein (ACP)] synthase III C terminal |
|  | LusiChr15G157800.1 | chr15:1342070:1346607 | 1212 | L.us.o.m.scaffold15.276 |  |
|  | LusiChr14G027370.1 | chr14:37021999:37025280 | 1227 | L.us.o.m.scaffold200.51 |  |
|  | LusiChr11G293930.1 | chr11:1968943:1972176 | 1227 | L.us.o.m.scaffold236.54 |  |
| KAS I | LusiChr9G104820.1 | chr9:7181622:7184957 | 1425 | L.us.o.m.scaffold50.105 | PF00109.29:Beta-ketoacyl synthase, N-terminal domain  PF02801.25:Beta-ketoacyl synthase, C-terminal domain |
|  | LusiChr14G018210.1 | chr14:32248377:32251546 | 1428 | L.us.o.m.scaffold4.384 |  |
|  | LusiChr13G427970.1 | chr13:22812085:22815087 | 1410 | L.us.o.m.scaffold36.59 |  |
|  | LusiChr4G323570.1 | chr4:3109013:3112302 | 1410 | L.us.o.m.scaffold115.44 |  |
|  | LusiChr15G155600.1 | chr15:320808:323654 | 1455 | L.us.o.m.scaffold76.25 |  |
|  | LusiChr13G409300.1 | chr13:290753:293815 | 1455 | L.us.o.m.scaffold15.61 |  |
|  | LusiChr11G298320.1 | chr11:4817438:4822720 | 1692 | L.us.o.m.scaffold9.103 |  |
|  | LusiChr9G118610.1 | chr9:31103902:31109871 | 1692 |  |  |
| KAS II | LusiChr11G298320.1 | chr11:4817438:4822720 | 1692 | L.us.o.m.scaffold9.103 |  |
|  | LusiChr9G118610.1 | chr9:31103902:31109871 | 1692 | L.us.o.m.scaffold248.18 |  |
|  | LusiChr9G104820.1 | chr9:7181622:7184957 | 1425 | L.us.o.m.scaffold50.105 |  |
|  | LusiChr14G018210.1 | chr14:32248377:32251546 | 1428 | L.us.o.m.scaffold4.384 |  |
|  | LusiChr4G323570.1 | chr4:3109013:3112302 | 1410 |  |  |
|  | LusiChr13G427970.1 | chr13:22812085:22815087 | 1410 |  |  |
| KAR | LusiChr9G097900.1 | chr9:3582031:3584285 | 972 | L.us.o.m.scaffold113.100 | PF00106.28: short chain dehydrogenase  PF08659.13: KR domain  PF13561.9: Enoyl-(Acyl carrier protein) reductase |
|  | LusiChr10G139960.1 | chr10:26605971:26608181 | 969 | L.us.o.m.scaffold59.257 |  |
| HAD | LusiChr11G310010.1 | chr11:28192757:28194174 | 483 | L.us.o.m.scaffold133.61 | PF01575.22:MaoC like domain |
|  | LusiChr10G147690.1 | chr10:30324182:30325402 | 498 | L.us.o.m.scaffold83.144 |  |
|  | LusiChr5G388800.1 | chr5:4001299:4001996 | 516 | L.us.o.m.scaffold88.197 |  |
|  | LusiChr8G054910.1 | chr8:32604855:32605318 | 366 | L.us.o.m.scaffold226.6 |  |
| ENR | LusiChr14G010420.1 | chr14:5294770:5296887 | 1170 | L.us.o.m.scaffold3.664 | PF00106.28: short chain dehydrogenase; PF13561.9: Enoyl-(Acyl carrier protein) reductase |
|  | LusiChr12G461460.1 | chr12:22373649:22376429 | 1149 | L.us.o.m.scaffold160.118 |  |
|  | LusiChr1G197320.1 | chr1:5037612:5040432 | 1173 | L.us.o.m.scaffold31.99 |  |
|  | LusiChr1G205440.1 | chr1:8973179:8975806 | 1236 |  | PF13561.9: Enoyl-(Acyl carrier protein) reductase |
| SAD | LusiChr2G067640.1 | chr2:2180027:2183116 | 1191 | L.us.o.m.scaffold68.112 | PF03405.17: Fatty acid desaturase |
|  | LusiChr3G375900.1 | chr3:26738240:26740758 | 1191 | L.us.o.m.scaffold13.424 |  |
|  | LusiChr6G229580.1 | chr6:1109653:1111624 | 1185 | L.us.o.m.scaffold205.47 |  |
|  | LusiChr1G225560.1 | chr1:31749361:31751435 | 1185 | L.us.o.m.scaffold194.104 |  |
| FAD | LusiChr12G461540.1 | chr12:22415655:22419265 | 1149 | L.us.o.m.scaffold3.651 | PF00487.27: Fatty acid desaturase |
|  | LusiChr14G010520.1 | chr14:5338358:5341783 | 1149 | L.us.o.m.scaffold31.110 |  |
|  | LusiChr7G264910.1 | chr7:2038533:2039669 | 1137 | L.us.o.m.scaffold28.441 |  |
|  | LusiChr1G197400.1 | chr1:5061635:5062753 | 1119 | L.us.o.m.scaffold3.656 |  |
|  | LusiChr14G010480.1 | chr14:5320542:5321991 | 1149 | L.us.o.m.scaffold3.654 |  |
|  | LusiChr12G461520.1 | chr12:22401838:22403308 | 1155 | L.us.o.m.scaffold3.655 |  |
|  | LusiChr14G010500.1 | chr14:5330275:5331417 | 1143 | L.us.o.m.scaffold117.66 |  |
|  | LusiChr14G010490.1 | chr14:5327114:5328427 | 1125 | L.us.o.m.scaffold169.100 |  |
|  | LusiChr1G204060.1 | chr1:8306768:8309422 | 1359 | L.us.o.m.scaffold253.14 |  |
|  | LusiChr1G195710.1 | chr1:4378585:4381159 | 1359 | L.us.o.m.scaffold84.251 |  |
|  | LusiChr13G432150.1 | chr13:25103180:25108174 | 1284 | L.us.o.m.scaffold41.105 |  |
|  | LusiChr4G319460.1 | chr4:1122720:1126299 | 1176 | L.us.o.m.scaffold45.90 |  |
|  | LusiChr4G327320.1 | chr4:4761358:4763732 | 1179 |  |  |
|  | LusiChr9G102630.1 | chr9:6052989:6055453 | 1179 |  |  |

**Table S20. Overlap of transposons and fatty acid pathway genes in the flax genome.**

| Gene Number | Transposon location | Target | Class | PercDiv | PercDel | PercIns |
| --- | --- | --- | --- | --- | --- | --- |
| LusiChr3G356900.1  (MCAT) | 3525900-3526136 | rnd-5_family-9933 | LTR/unknown | 26.1 | 11.0 | 6.0 |
| LusiChr13G411420.1  (KAS III) | 1200681-1200767 | rnd-2_family-186 | DNA/unknown | 25.4 | 0.0 | 4.8 |
| LusiChr9G104820.1  (KAS I/II) | 7182464-7182540 | rnd-1_family-23 | LTR/unknown | 16.9 | 0.0 | 0.0 |
|  | 7183779-7183965 | rnd-2_family-186 | DNA/unknown | 15.0 | 4.3 | 0.0 |
| LusiChr15G155600.1  (KAS I/II) | 320974-321146 | rnd-2_family-195 | SINE/unknown | 24.4 | 0.6 | 0.6 |
| LusiChr13G409300.1  (KAS I/II) | 292604-292796 | rnd-4_family-746 | LTR/unknown | 27.3 | 0.5 | 3.2 |
|  | 292799-292945 | rnd-4_family-425 | DNA/unknown | 18.1 | 10.9 | 2.5 |
| LusiChr11G298320.1  (KAS I/II) | 4821806-4821881 | Copia-45_AIp-I | LTR/Copia | 26.3 | 2.6 | 0.0 |
|  | 4822616-4822717 | rnd-5_family-7954 | LTR/unknown | 22.6 | 4.9 | 0.0 |
|  | 4822651-4822791 | rnd-5_family-12052 | DNA/unknown | 17.8 | 0.0 | 4.4 |
| LusiChr9G118610.1  (KAS I/II) | 31103901-31103975 | MuDR-N3_ATr | DNA/MULE-MuDR | 20.0 | 4.0 | 6.8 |
|  | 31103912-31103973 | DNA3-13B_CGi | DNA/PIF-Harbinger | 15.0 | 4.8 | 3.2 |
|  | 31103939-31104025 | hAT-1_NN | DNA/hAT-Ac | 19.5 | 10.3 | 0.0 |
|  | 31103974-31103991 | DNA3-10_CGi | DNA | 14.1 | 0.0 | 11.1 |
|  | 31104026-31104055 | LTR42_MD | LTR/ERV1 | 21.9 | 2.6 | 6.2 |
|  | 31108542-31108617 | Copia-45_AIp-I | LTR/Copia | 26.3 | 2.6 | 0.0 |
| LusiChr1G197320.1  (ENR) | 5038671-5038760 | rnd-1_family-69 | LTR/unknown | 10.2 | 6.7 | 2.1 |
| LusiChr1G205440.1  (ENR) | 8974080-8974093 | rnd-1_family-69 | LTR/unknown | 10.8 | 1.9 | 4.8 |
|  | 8974094-8974234 | rnd-1_family-86 | DNA/unknown | 16.9 | 0.0 | 3.7 |
| LusiChr2G067640.1  (SAD) | 2180334-2180497 | rnd-1_family-31 | Unknown | 19.5 | 7.9 | 2.9 |
|  | 2180479-2180557 | rnd-1_family-65 | DNA/unknown | 11.4 | 0.0 | 0.0 |
|  | 2180559-2180755 | rnd-1_family-33 | DNA/unknown | 17.1 | 2.5 | 0.2 |
|  | 2182019-2182142 | rnd-5_family-675 | DNA/unknown | 11.3 | 0.8 | 0.0 |
|  | 2182196-2182355 | rnd-4_family-1387 | DNA/unknown | 24.6 | 6.2 | 3.7 |
|  | 2182728-2182823 | rnd-2_family-186 | DNA/unknown | 22.0 | 5.2 | 5.2 |
| LusiChr6G229580.1  (SAD) | 1110937-1111056 | rnd-3_family-346 | DNA/unknown | 20.8 | 1.7 | 0.0 |
|  | 1111051-1111139 | rnd-3_family-346 | DNA/unknown | 17.0 | 0.0 | 1.1 |
| LusiChr1G225560.1  (SAD) | 31749864-31750142 | rnd-3_family-346 | DNA/unknown | 17.0 | 7.5 | 1.0 |
| LusiChr12G461540.1  (FAD) | 22417811-22418233 | rnd-5_family-13269 | LINE/L1 | 20.6 | 1.4 | 0.0 |
|  | 22418230-22418914 | rnd-5_family-13269 | LINE/L1 | 24.0 | 0.6 | 0.1 |
| LusiChr1G204060.1  (FAD) | 8307001-8307039 | rnd-5_family-12846 | LTR/unknown | 7.7 | 0.0 | 0.0 |
|  | 8307398-8307534 | rnd-5_family-13269 | LINE/L1 | 32.1 | 2.2 | 0.0 |
| LusiChr1G195710.1  (FAD) | 4378740-4378797 | rnd-5_family-12846 | LTR/unknown | 4.2 | 0.0 | 18.4 |
|  | 4379134-4379270 | rnd-5_family-13269 | LINE/L1 | 32.9 | 2.2 | 0.0 |
| LusiChr13G432150.1  (FAD) | 25103441-25103978 | rnd-2_family-202 | LINE/unknown | 17.7 | 11.9 | 4.3 |
|  | 25104860-25104981 | rnd-3_family-535 | DNA/unknown | 9.7 | 5.7 | 6.6 |
| LusiChr4G319460.1  (FAD) | 1124168-1124311 | rnd-3_family-533 | Unknown | 31.0 | 9.0 | 1.3 |
|  | 1125162-1125757 | rnd-2_family-202 | LINE/unknown | 17.5 | 3.4 | 10.0 |
| LusiChr4G327320.1  (FAD) | 4761901-4762019 | rnd-5_family-13269 | LINE/L1 | 31.1 | 2.5 | 0.0 |
| LusiChr9G102630.1  (FAD) | 6054668-6054786 | rnd-5_family-13269 | LINE/L1 | 29.4 | 2.5 | 0.0 |

Note: PercDiv, per centage of divergence; PercDel, per centage of delete; PercIns, per centage of insert

**Table S21. The information of identified 15 motifs in SAD and FAD proteins**

| **Motif name** | **Motif sequence** | **width** | **E-value** | **Sites** | **Function** |
| --- | --- | --- | --- | --- | --- |
| Motif1 | GFILHSSJLVPYFSWKISHRRHHQNTGSLERDE | 33 | 2.1e-291 | 14 | Fatty acid desaturase |
| Motif2 | NNVFHNITDTHVAHHLFSTIPHYHAMEATKAIKPVLGEYYR | 41 | 2.9e-375 | 14 | Fatty acid desaturase |
| Motif3 | YVAWPLYWAAQGTMFTAVWVLGHECGHHAFSD | 32 | 3.2e-278 | 14 | Fatty acid desaturase |
| Motif4 | HTHPKLPWYRSSEWDWLRGALATVDRDYG | 29 | 4.3e-238 | 14 | Fatty acid desaturase |
| Motif5 | KRDPSSKPPFTLADIKKAIPPHCFKRSIPRSFSYVAYDLTV | 41 | 9.3e-303 | 14 | Fatty acid desaturase |
| Motif6 | RPYGKFASHFBPYSPJYNDRERKEVFLSD | 29 | 8.3e-171 | 14 |  |
| Motif7 | KQKSEIGWYSKYLNNPPGRVLTLAVTLTLGWPLYLAFNVSG | 41 | 4.7e-145 | 8 |  |
| Motif8 | VYGVPLLIVNGWLDLITYLH | 20 | 3.3e-114 | 14 |  |
| Motif9 | DGTPFIKALWREAKECVYVEPDEGDQNKG | 29 | 1.4e-111 | 8 |  |
| Motif10 | RVDMRQIEKTIQYLIGSGMDPGTENNPYLGFIYTSFQERATFISHGNTAR | 50 | 3.8e-108 | 4 | Fatty acid desaturase, type 2 |
| Motif11 | VGDMITEDALPTYZTMJNTLDGVRDETGASQSPWAJWTRAWTAEENRHGD | 50 | 1.6e-103 | 4 | Fatty acid desaturase, type 2 |
| Motif12 | MMRKKISMPAHLMYDGEDPKLFDNYSSVAQRJGVYTAKDYADILEFLIGR | 50 | 7.4e-94 | 4 | Fatty acid desaturase, type 2 |
| Motif13 | EPKKSGPFPFHLFGDLVRSLGEDHYVSDTGDVVFYQSDPEI | 41 | 1.8e-116 | 6 |  |
| Motif14 | KVYKSLDTSTKFMRFTIPLPMFAYPIYLW | 29 | 3.2e-80 | 6 |  |
| Motif15 | LKHQQKTHSMPPZKJEJFKSLEGWAEDNLLPHLKPVEKCWQPQDFLPDPE | 50 | 1.4e-77 | 4 | Fatty acid desaturase, type 2 |

**Table S22. The list primer was used for gene expression analysis by qRT-PCR.**

| Gene ID | Forward primer | Reverse primer |
| --- | --- | --- |
| LusiChr9G109570.1 | CAGGTCAACTAAACGGCATTA | TGGCAGCATACTCGATCAAAC |
| LusiChr14G010420.1 | TGCGTCTATGACCAGGCTTAC | GGGCTAGTCTCGCTTCCAG |
| LusiChr12G461460.1 | TTGACGAATCGCGTGTATT | GCATATCGCTTGTTTGTTTT |
| LusiChr1G197320.1 | GTCTTACCCGACGGTTCTC | GCTCCTTAACGGTCCAATT |
| LusiChr1G205440.1 | AGTTACTCATTCATTTCCCTC | GCTCATCCCACCACCATAA |
| LusiChr12G461540.1 | TGCCGCCATCTTCTACTACAT | GTGGGCTAGGACCCAGACA |
| LusiChr14G010520.1 | TTGCCGCACTACAAATCCT | CGTGGGTATCCGTGATGTTA |
| LusiChr7G264910.1 | TACTTCCACCTCCTCCCTA | TGAGCCAACACCCATACTC |
| LusiChr1G197400.1 | TACTTCCACCTCCTCCCTA | TGAGCCAACACCCATACTC |
| LusiChr14G010480.1 | GCTTCTGGCACTGGCTTAC | GGACATCGGGTAGAAATGG |
| LusiChr12G461520.1 | TCAGTTGCGGTGGTGGTAC | CGAGGAGGATGGTGTAAGC |
| LusiChr14G010500.1 | CTCAGCCCTCCTTTCACCT | TTGGGTTTAGGGACGAACA |
| LusiChr14G010490.1 | TACCACCATAAAGCGAACA | CGTAGGCTAGGTAGGTGAAG |
| LusiChr1G204060.1 | TGGTTGGATTTCGTAACTT | AATATCGTGGTGGATGTTG |
| LusiChr1G195710.1 | GACGCATCATCAGAACCAT | GAAAGGCAATGTGAACCTC |
| LusiChr13G432150.1 | AGAACCGAAGAAATCAGGG | TTGGGAATATGTGGGTCAG |
| LusiChr4G319460.1 | AGAGCCGAAGAAATCAGGG | GGTACGGAACTTGGGAATA |
| LusiChr4G327320.1 | TATACCGTCCCTTACCTGA | CTCTTTGCCTCTGTACCAC |
| LusiChr9G102630.1 | GCTCTACTGGATCGCTCAG | GTAAGGCACAAGGATTGCA |
| LusiChr11G310010.1 | ATCTCGTCTCACTTCCCTG | TTTGGCGATGTACTTGTTA |
| LusiChr10G147690.1 | TCCCTTCTGTAATGGTGGC | TCCGGTTCTAAGTGGTGGA |
| LusiChr5G388800.1 | GAATAACAAGCACATCGCTAA | CACCTTCAAAGAGGGCAAA |
| LusiChr8G054910.1 | GCTACTATCCCACCACTTA | GATGCAGAGGGTTCAAATC |
| LusiChr9G104820.1 | CTTTGCTGGCGATTGAACT | CCTCGGCGAATATGGTTAG |
| LusiChr14G018210.1 | CGCCAGGAGGAGAACAACT | CATGCCCGTGATTACGACT |
| LusiChr13G427970.1 | CTTGGCGTTTCTACTTGTAT | CCTTCTTTATGGCATTTATC |
| LusiChr4G323570.1 | TAGTGCTCAGGGATACATCG | CTCCAGCTTTCTGCAAGTCAA |
| LusiChr15G155600.1 | TTACGGGAATGGGATTGGT | ATCGGACGGAGAAAGTGGA |
| LusiChr13G409300.1 | TGGAGGATGCTAAAGTATCTC | TTCCGTTCATCTTCATCTCG |
| LusiChr11G298320.1 | CTAAGGGTCTCGTACAGAAA | AGCTGTCGAAATGGAATAG |
| LusiChr9G118610.1 | CCTCACGACCTTGGGATAA | ATGTTGGCACCTCTGCTCT |
| LusiChr11G298320.1 | CTAAGGGTCTCGTACAGAAA | AGCTGTCGAAATGGAATAG |
| LusiChr9G118610.1 | CCTCACGACCTTGGGATAA | ATGTTGGCACCTCTGCTCT |
| LusiChr9G104820.1 | CTTTGCTGGCGATTGAACT | CCTCGGCGAATATGGTTAG |
| LusiChr14G018210.1 | CGCCAGGAGGAGAACAACT | CATGCCCGTGATTACGACT |
| LusiChr4G323570.1 | TAGTGCTCAGGGATACATCG | CTCCAGCTTTCTGCAAGTCAA |
| LusiChr13G427970.1 | CTTGGCGTTTCTACTTGTAT | CCTTCTTTATGGCATTTATC |
| LusiChr13G411420.1 | GGTATCAGTGGATCTGGGTT | TTGCAGCCTTGACTAACAA |
| LusiChr15G157800.1 | GTTCTAACGGATCGGTTCA | CGAGGGAGGATTCTATGGT |
| LusiChr14G027370.1 | ATGAACGGGAAAGAGGTAT | GTCTATGTTGGCTGAGGTT |
| LusiChr11G293930.1 | TCGGAGTGGTAAGGTGAAG | CATCTAATGATAGCAGAGCC |
| LusiChr3G356900.1 | GCGGATTTGGTGTCAAGAG | TGTCCAGGGAACAAGAAGG |
| LusiChr1G193830.1 | TCCTGCCACGATTAAGAAA | CGGGTCCTAACTCATAGCT |
| LusiChr2G067640.1 | TGCCGACTTACCAGACAAT | GGTCACCGTGCCTATTCTC |
| LusiChr3G375900.1 | ATAGGCACGGTGACCTTCT | CAGAGCCGATGAGATACTGA |
| LusiChr6G229580.1 | CAAATGCTGGCAGCCTCAG | CCCACCAAGACGACGAAGTA |
| LusiChr1G225560.1 | AAGCCCGTCAATAAATGCT | TCCTTTCCCTCAACAACCT |
| LusiChr9G097900.1 | ATCTGCCGTGTAAGGTGAA | CGAGGGAGGATAGATACTG |
| LusiChr10G139960.1 | AGGCACGGTGGGGATAGTG | CATCGGGTAGCAAGTGGTG |
